# Effects of exercise on pain, fatigue, and quality of life in people with fibromyalgia: a systematic review and meta-analysis of randomized controlled trials

**DOI:** 10.3389/fmed.2026.1782714

**Published:** 2026-02-18

**Authors:** Tongling Wang, Hao Su, Yilun Zhou, Liwen Du, Yuanyuan Lv, Laikang Yu

**Affiliations:** 1 School of Sports Science, Nantong University, Nantong, China; 2 Beijing Key Laboratory of Sports Performance and Skill Assessment, Beijing Sport University, Beijing, China; 3 Department of Strength and Conditioning Assessment and Monitoring, Beijing Sport University, Beijing, China; 4 China Institute of Sport and Health Science, Beijing Sport University, Beijing, China

**Keywords:** exercise, fatigue, fibromyalgia, meta-analysis, pain, quality of life, systematic review

## Abstract

**Background:**

An increasing number of studies have explored the effects of exercise on pain, fatigue, and quality of life (QOL) in fibromyalgia patients, yet the available results remain inconsistent. This study aimed to examine the effects of exercise on pain, fatigue, and QOL in fibromyalgia patients.

**Methods:**

A comprehensive literature search, restricted to studies published up to 30 July 2025, was conducted across five databases: Embase, PubMed, Cochrane Library, Web of Science, and Scopus. The search utilized the following keywords: exercise, fibromyalgia, pain, fatigue, and quality of life. A meta-analysis was performed to calculate the standardized mean difference (SMD) and 95% confidence interval.

**Results:**

Twenty-four studies were included in this meta-analysis. Exercise had a positive effect on alleviating pain (SMD, −0.77; *p* < 0.00001), fatigue (SMD, −0.39; *p* = 0.03), and QOL (SMD, 0.53; *p* < 0.00001) in fibromyalgia patients. Subgroup analyses showed that aerobic exercise (SMD, −0.83; *p* = 0.0002) conducted for ≥8 weeks (SMD, −0.73; *p* = 0.006), <3 times per week (SMD, −1.12; *p* = 0.0009), 60–90 min per session (SMD, −1.35; *p* = 0.0001), and <180 min per week (SMD, −0.96; *p* = 0.001) were more effective in alleviating pain.

**Conclusion:**

Exercise significantly improved pain, fatigue, and QOL in fibromyalgia patients. To alleviate pain, fibromyalgia patients are recommended to engage in aerobic exercise for at least 8 weeks, 60–90 min per session, twice weekly, without exceeding 180 min weekly.

**Systematic review registration:**

https://www.crd.york.ac.uk/PROSPERO/view/CRD42023493753.

## Introduction

Fibromyalgia represents a widespread chronic condition characterized by sustained, non-inflammatory musculoskeletal pain (1). Beyond pain, patients frequently exhibit associated manifestations such as pronounced fatigue, disrupted sleep patterns, and heightened levels of psychological distress, including anxiety (2–4). This diverse range of symptoms significantly impairs patients’ quality of life (QOL) (5, 6), affecting both physical functioning and emotional well-being. Individuals with fibromyalgia often have poor tolerance for high-intensity physical activity, leading to a sedentary lifestyle (7). Combined with negative psychological states, this lifestyle may exacerbate fibromyalgia symptoms and increases the risk of additional comorbidities (8, 9). The general population’s fibromyalgia prevalence is estimated between 2 and 4% (1), with women being three times more likely to be affected than men (10). Notably, higher prevalence rates have been reported predominantly in developed countries, such as Spain (2.4%), Germany (3.2%), and Italy (3.6%), which may partly reflect greater research capacity and scientific output, whereas epidemiological data from low- and middle-income countries remain scarce (11).

The pathogenesis of fibromyalgia remains unclear, but central sensitization, peripheral sensitization, and inflammatory/immune mechanisms are widely recognized (12). These mechanisms lower the pain threshold, increase pain sensitivity, and create a bidirectional relationship between pain and emotional distress. Given fibromyalgia’s complex pathophysiology and pharmacological treatments’ potential side effects (12–15), exercise therapy is considered a key management component. Evidence indicates that it alleviates pain, improves sleep, reduces fatigue and depressive symptoms (16–19), and ultimately contributes to better QOL in patients. For example, aerobic exercise interventions have been shown to reduce pain in women with fibromyalgia while concurrently improving flexibility, balance, and overall QOL (20). Likewise, Baduanjin and other forms of aerobic exercise have demonstrated efficacy in alleviating pain and fatigue, reducing depressive symptoms, and enhancing QOL (21). Resistance exercise has also been reported to alleviate mental and physical fatigue (22) and significantly improve pain, psychological well-being, and QOL (23). Virtual reality-assisted exercise further demonstrates benefits in reducing pain and fatigue while enhancing aerobic capacity and QOL in fibromyalgia patients (24, 25). Nevertheless, exercise responses appear heterogeneous, and excessive or poorly tailored exercise may exacerbate symptoms, potentially leading to overtraining-related impairments in physical and psychological health (26, 27).

Although multiple systematic reviews and meta-analyses have examined the effects of exercise in fibromyalgia, several critical gaps remain. Most prior meta-analyses (28, 29) have evaluated exercise effects on isolated outcomes, such as pain or fatigue, without sufficiently considering the interrelationships among core symptom domains. Pain, the defining feature of fibromyalgia, is closely intertwined with fatigue, psychological distress, and QOL, and these symptoms may mutually reinforce one another (30). However, existing evidence syntheses have rarely explored how exercise-related improvements in pain relate to changes in other clinically relevant outcomes, thereby limiting their translational value for individualized exercise prescription.

In addition, substantial methodological limitations characterize the existing literature. Several reviews included studies that failed to report key components of exercise interventions, such as intensity, frequency, or session duration (28, 29, 31), while others relied on broadly defined intervention durations. For example, one study (32) concluded that exercise improved fibromyalgia symptoms but recommended a wide intervention window of 13–24 weeks, offering limited practical guidance for clinical decision-making. A further meta-analysis (33) categorized interventions into circuit-based and exercise-movement technique modalities (e.g., Tai Chi and Yoga); however, these classifications encompass overlapping physiological mechanisms, potentially obscuring meaningful dose–response relationships and diminishing clinical interpretability. Moreover, the lack of integrated analyses examining how different outcomes respond to varying exercise prescriptions constrains the applicability of these findings. For instance, the exercise dose required to alleviate pain may differ from that needed to meaningfully improve QOL, yet this distinction has not been systematically addressed. Another limitation of prior syntheses is the exclusion of several classical and widely used exercise modalities, including Tai Chi, Qigong, and Yoga, in some analyses (31, 34), despite their relevance and acceptability in fibromyalgia management.

In light of these gaps, the present systematic review and meta-analysis aimed to provide a more comprehensive and integrative evaluation of exercise interventions in fibromyalgia. The primary objective was to quantify the effects of exercise on pain, fatigue, and QOL in fibromyalgia patients. The secondary objectives were to explore the optimal type of exercise, intervention duration, frequency, session duration, and weekly time in fibromyalgia patients.

## Methods

### Design

This study was conducted in accordance with the Preferred Reporting Items for Systematic Reviews and Meta-Analyses (PRISMA) guidelines (35), thereby ensuring methodological rigor and transparency in reporting. The study protocol was prospectively registered in PROSPERO under the identifier CRD42023493753.

### Search strategy

A systematic search was performed to identify eligible randomized controlled trials (RCTs). Five databases (Embase, PubMed, Cochrane Library, Web of Science, and Scopus) were searched for relevant studies published up to July 30, 2025. Search terms included both keywords and Medical Subject Headings (MESH), covering concepts such as exercise, fibromyalgia, pain, fatigue, and quality of life (Supplementary Table S1). In addition, reference lists of the retrieved articles were manually checked to capture further studies not identified in the initial search. Two authors (TW and HS) independently screened and selected the studies. Any discrepancies were resolved through discussion with a third author (LY) until consensus was achieved.

### Inclusion and exclusion criteria

The inclusion criteria were formulated according to the Population, Intervention, Comparison, and Outcome (PICO) principle: (1) Population: individuals diagnosed with fibromyalgia; (2) Intervention: RCTs in which participants were randomly allocated to an intervention or control group; (3) Comparison: trials reporting baseline and post-intervention assessments of pain, fatigue, or QOL; and (4) Outcome: the primary endpoint was pain, while fatigue and QOL were considered secondary outcomes.

Exclusion criteria were: (1) articles not published in English; (2) studies reporting results in a format that could not be converted to mean ± standard deviation (SD); and (3) trials without a control group.

### Data extraction

Two authors (TW and HS) extracted data using a pre-specified template. Extracted information included: (1) the first author and year of publication; (2) sample size, age, and gender distribution; (3) intervention type, duration, session duration, frequency, and weekly time; and (4) outcome measures related to pain, fatigue, and QOL. For all studies meeting the inclusion criteria, the above data were extracted. In cases where data were incomplete (except for outcome measures), only the reported data were extracted. Disagreements were resolved in consultation with a third author (LY).

### Methodological quality assessment

The risk of bias was independently appraised by two authors (TW and HS), with inconsistencies resolved through discussion. The Cochrane Risk of Bias tool (RoB) tool was applied (36, 37), which evaluates six methodological domains: random sequence generation, allocation concealment, blinding, incomplete outcome data, selection of outcome reports, and other biases. Each domain was classified as “low,” “high,” or “unclear” (38). Given the inherent limitations of the RoB (39), two independent authors (TW and HS) evaluated the overall evidence quality using GRADEpro GDT (Evidence Prime Inc., McMaster University, 2020) to generate summary of findings tables. To ensure objectivity, a third author (LY) arbitrated any discrepancies that arose during the assessment process.

### Statistical analysis

Given the variation in outcome measures for pain, fatigue, and QOL, effect sizes were synthesized using a random-effects model and expressed as standardized mean differences (SMD) with 95% confidence interval (CI). Statistical heterogeneity was quantified with the *I*^2^ statistic, where values of 0, 25, 50, and 75% indicated no, low, moderate, and high heterogeneity, respectively (31, 32). If heterogeneity was high (*I*^2^ > 60%), subgroup analysis and sensitivity analysis were conducted to explain the results (33, 34). Publication bias was assessed visually through funnel plots and formally tested using Egger’s test (35).

Subgroup analyses were stratified by intervention type (aerobic, resistance, multicomponent), intervention duration (<8 weeks, ≥8 weeks), training frequency (<3 times per week, ≥3 times per week), session duration (<60 min, 60–90 min), and weekly time (<180 min, ≥180 min). Forest plots were generated using RevMan 5.4 software, while sensitivity analysis, Egger’s test, and funnel plots were performed with Stata 17. Statistical significance was set at *p* < 0.05.

## Results

### Study selection

**Figure 1:**
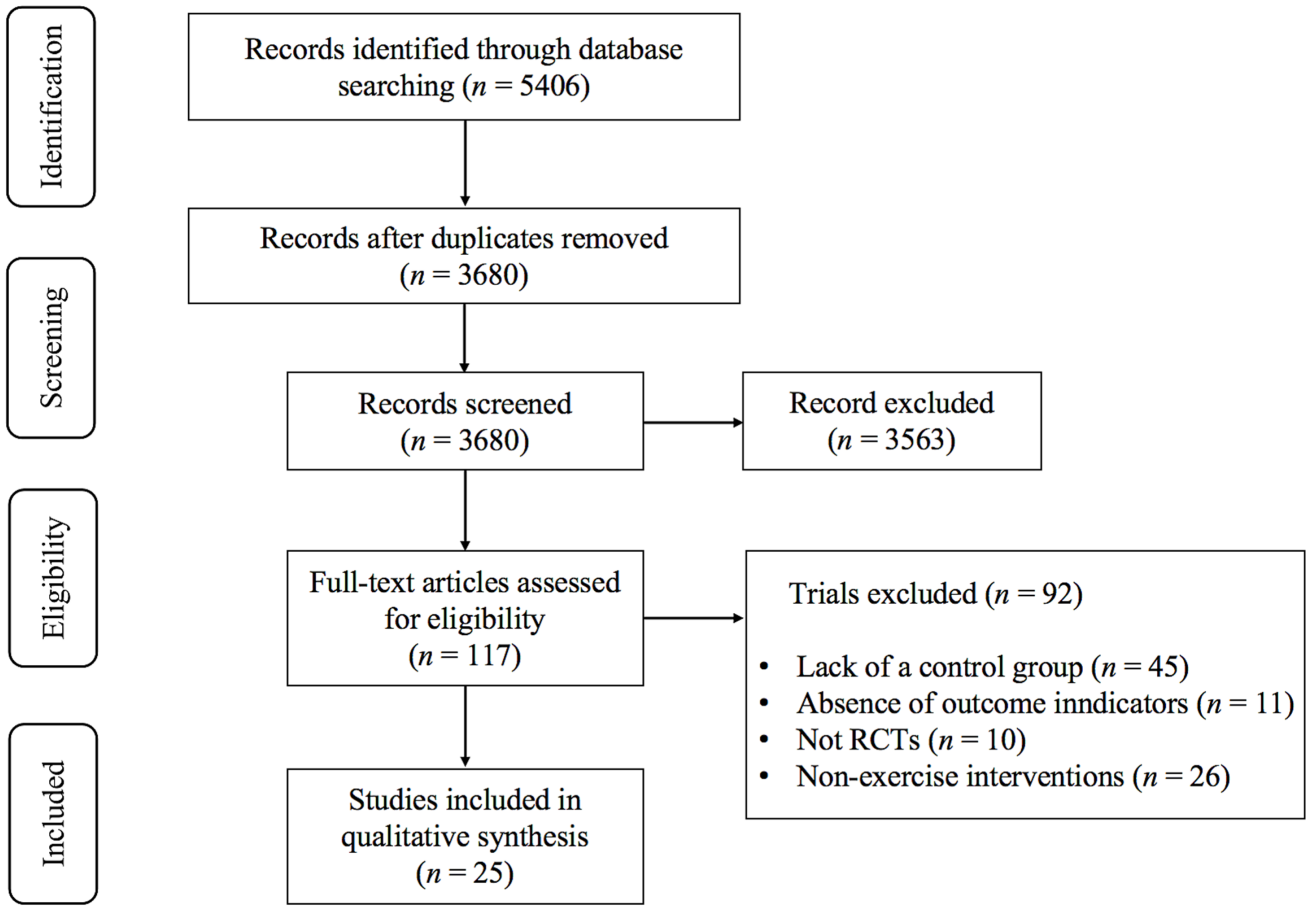
PRISMA flowchart of study selection.

Based on the literature search strategy, a total of 5,406 records were identified in the initial search. After removing 1726 duplicate records, 3,680 documents remained for screening. Title and abstract assessment led to the exclusion of 3,563 studies that did not satisfy the eligibility criteria. One hundred and seventeen studies were then subjected to full-text evaluation, of which 92 were excluded for various reasons. Ultimately, 25 studies fulfilled the inclusion criteria and were incorporated into this systematic review and meta-analysis. The overall selection process is summarized in the PRISMA flow diagram (Figure 1).

### Characteristics of the included studies

This study encompassed 25 studies (20–23, 25, 27, 40–58) involving 1740 fibromyalgia patients (Supplementary Table S2). The intervention group totaled 896 patients, 98.3% of whom were female. The exercise programs comprised aerobic exercise, resistance exercise, and multicomponent training, the latter integrating multiple physical capacities within a single session (58). The interventions lasted between 4 and 24 weeks, with individual sessions ranging from 3 to 90 min, and were conducted at a frequency of one to five times per week. The control group consisted of 844 patients, 97.8% female, who received non-exercise interventions such as conventional therapy, home care, relaxation stretching therapy, or no intervention. All included studies were RCTs and assessed at least one outcome indicator (i.e., pain, fatigue, or QOL). The meta-analysis included only outcomes assessed immediately after the intervention, excluding those assessed during post-intervention follow-up.

### Meta-analysis results

#### Effects of exercise on pain in fibromyalgia patients

**Figure 2:**
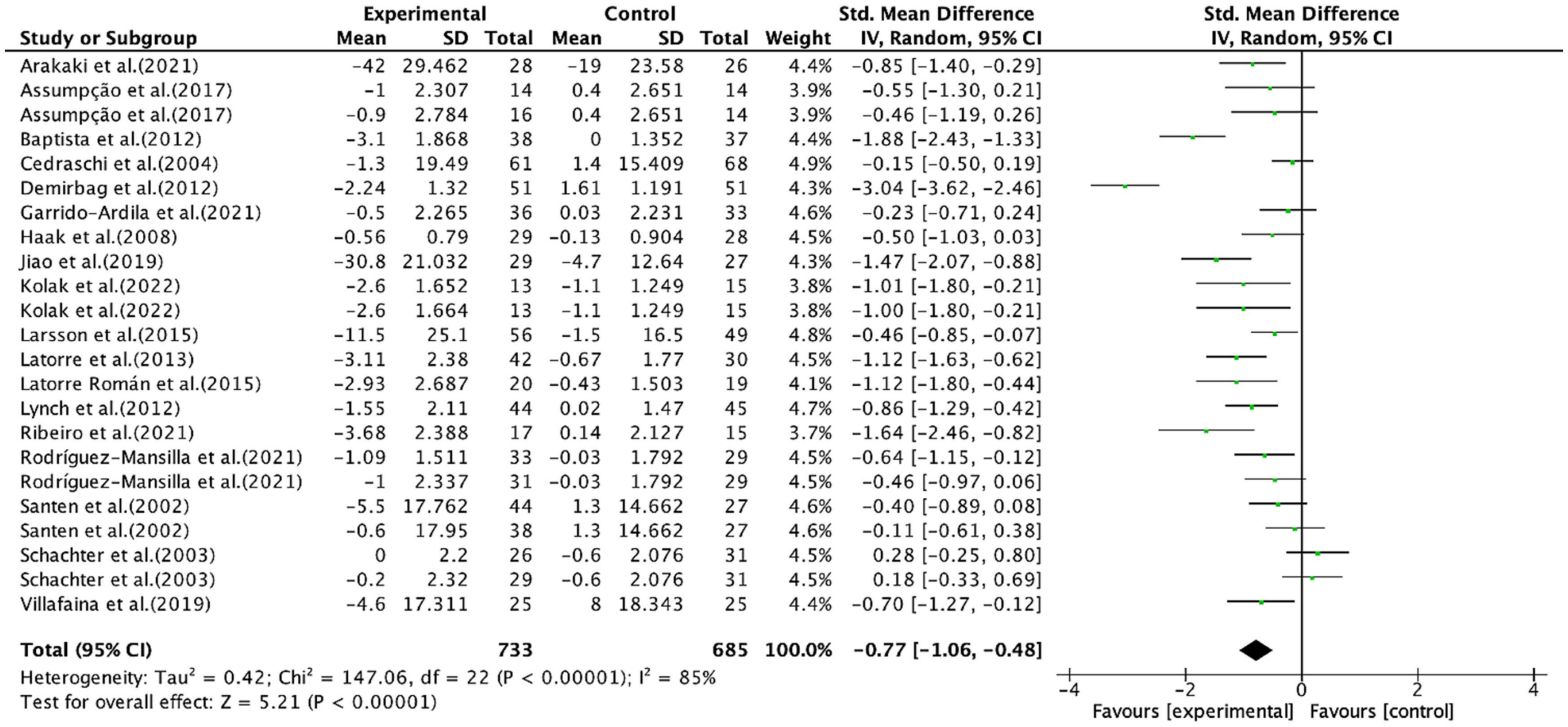
Meta-analysis results of the effects of exercise on pain in fibromyalgia patients.

A total of 18 studies (20, 21, 23, 27, 40–43, 45–52, 56, 57), covering 23 trails and 1,418 patients, provided data for pain, with 733 patients in the intervention group and 685 patients in the control group. Pain was predominantly assessed using the visual analog scale (VAS, 15 studies) (20, 21, 23, 27, 40, 42, 43, 45, 47–50, 52, 56, 57), regional pain score (RPS, 1 study) (42), verbal numeric scale (VNS, 1 study) (46), and numerical rating scale for pain intensity (NRS-PI, 1 study) (51). As depicted in Figure 2, exercise positively alleviated pain in fibromyalgia patients (SMD, −0.77; 95% CI, −1.06 to −0.48; *p* < 0.00001; *I*^2^ = 85%).

#### Effects of exercise on fatigue in fibromyalgia patients

**Figure 3:**
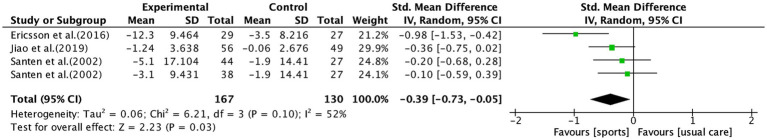
Meta-analysis results of the effects of exercise on fatigue in fibromyalgia patients.

Three studies (21, 22, 56), covering 4 trials and 297 patients, provided data for fatigue, with 167 patients in the intervention group and 130 patients in the control group. Fatigue was primarily assessed via the VAS (1 study) (56), multidimensional assessment of fatigue scale (MAF, 1 study) (21), multidimensional fatigue inventory (4–20) scale (MFI-20, 1 study) (22). As shown in Figure 3, exercise was beneficial for reducing fatigue in fibromyalgia patients (SMD, −0.39; 95% CI, −0.73 to −0.05; *p* = 0.03; *I*^2^ = 52%).

#### Effects of exercise on QOL in fibromyalgia patients

**Figure 4:**
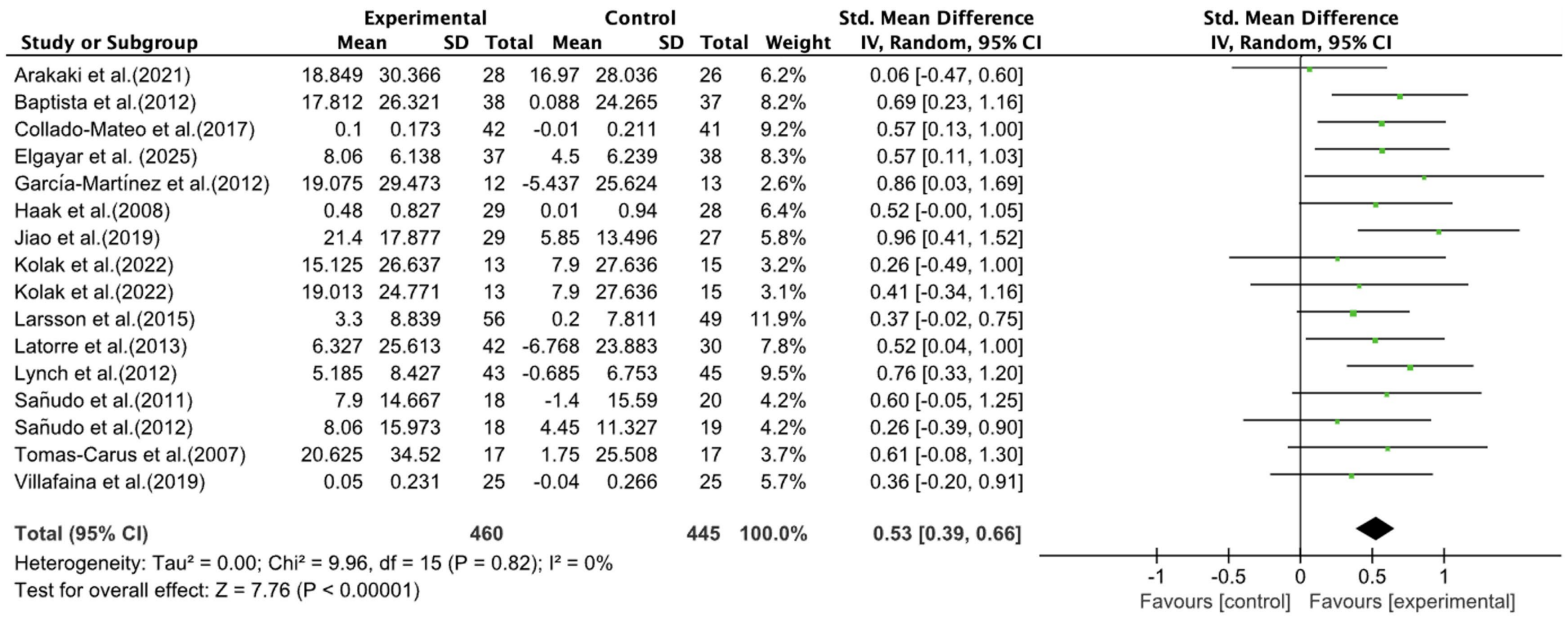
Meta-analysis results of the effects of exercise on quality of life in fibromyalgia patients.

Fifteen studies (21, 25, 40, 41, 44, 46–49, 51, 53–55, 57, 58), covering 17 trials and 905 patients, provided data for QOL, with 460 patients in the intervention group and 4 patients in the control group. QOL was mainly assessed using the short form health survey 36 scale (SF-36, 11 studies) (21, 40, 41, 44, 47–49, 51, 53–55), euroqol-5 dimensions-5 levels (EQ-5D-5L, 2 studies) (25, 57), the World Health Organization quality of life BREF (WHOQOL-BREF, 1 study) (46) and the 12-Item Short Form Health Survey (58). As illustrated in Figure 4, exercise positively enhanced QOL in fibromyalgia patients (SMD, 0.53; 95% CI, 0.39 to 0.66; *p* < 0.00001; *I*^2^ = 0%).

### Subgroup analysis

Given the high heterogeneity in pain (*I*^2^ = 85%), subgroup analyses were performed to explore optimal exercise regimens.

**Figure 5:**
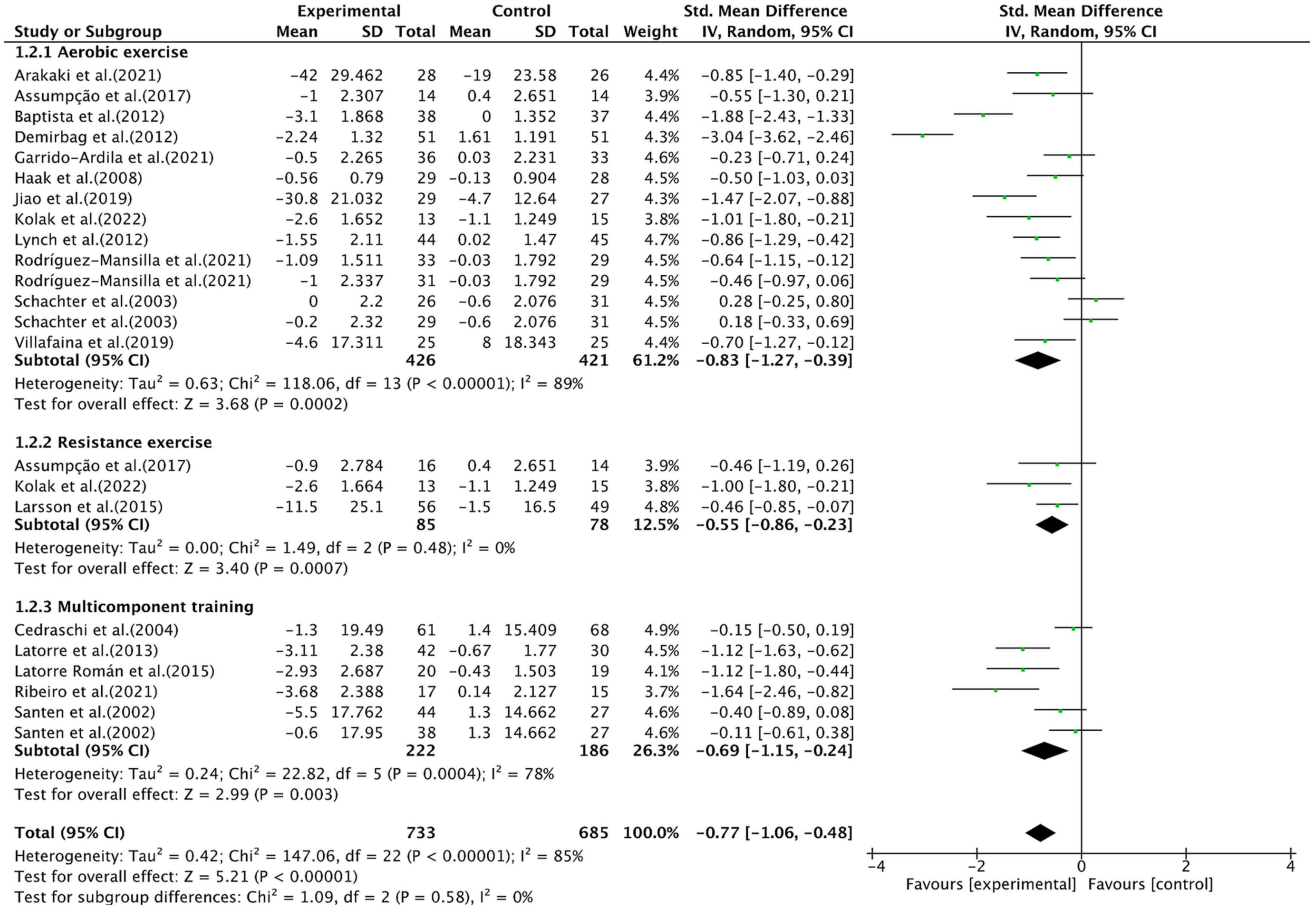
Subgroup analysis by intervention type (aerobic exercise, resistance exercise, multicomponent training) on pain outcomes.

Aerobic exercise (SMD, −0.83; 95% CI, −1.27 to −0.39; *p* = 0.0002; *I*^2^ = 89%), resistance exercise (SMD, −0.55; 95% CI, −0.86 to −0.23; *p* = 0.0007; *I*^2^ = 0%), and multicomponent training (SMD, −0.69; 95% CI, −1.15 to −0.24; *p* = 0.003; *I*^2^ = 78%) all significantly alleviated pain in fibromyalgia patients (Figure 5), with aerobic exercise proving the most effective. Further subgroup analyses of aerobic exercise were conducted.

**Figure 6:**
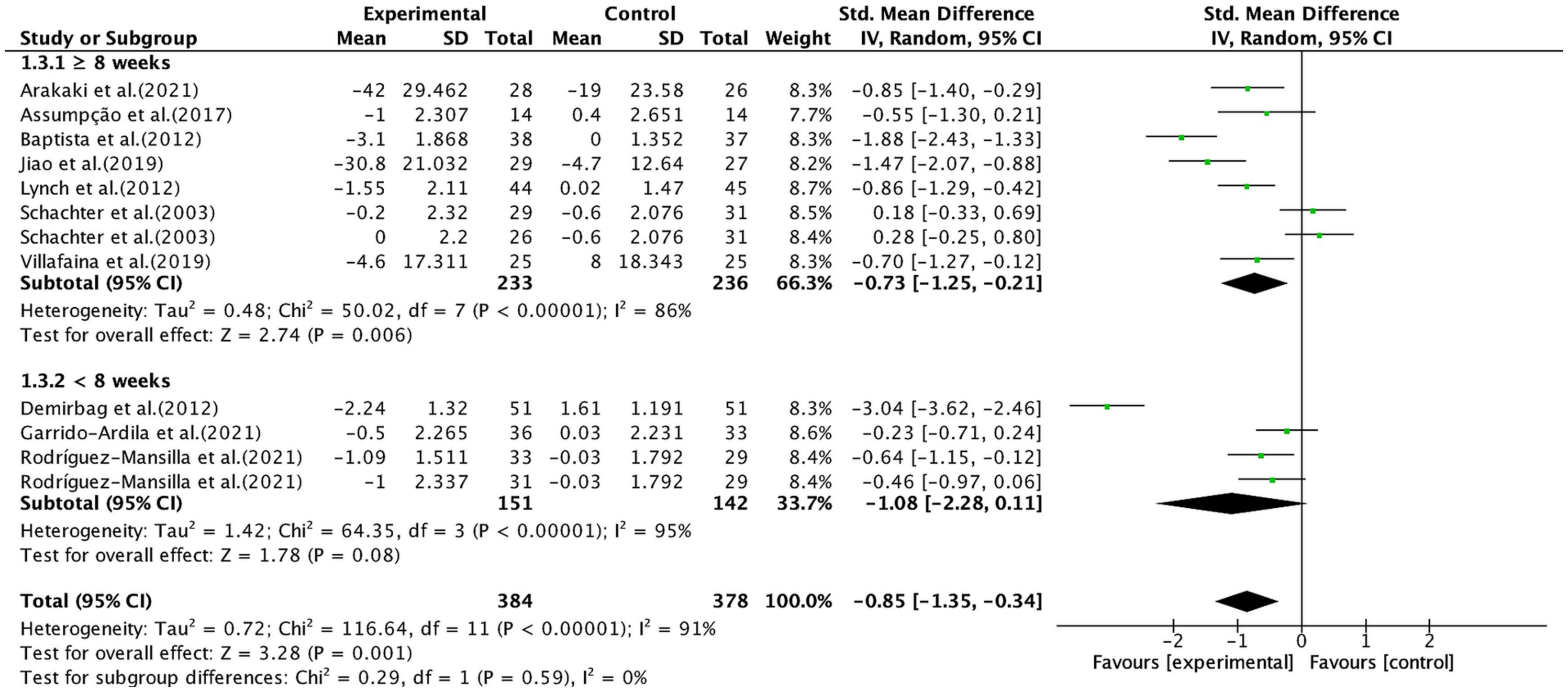
Subgroup analysis by aerobic exercise duration (<8 weeks, ≥8 weeks) on pain outcomes.

As shown in Figure 6, ≥8 weeks of aerobic exercise significantly alleviated pain (SMD, −0.73; 95% CI, −1.25 to −0.21; *p* = 0.006; *I*^2^ = 86%), whereas <8 weeks of aerobic exercise showed no significant effect (SMD, −1.08; 95% CI, −2.28 to 0.11; *p* = 0.08; *I*^2^ = 95%).

**Figure 7:**
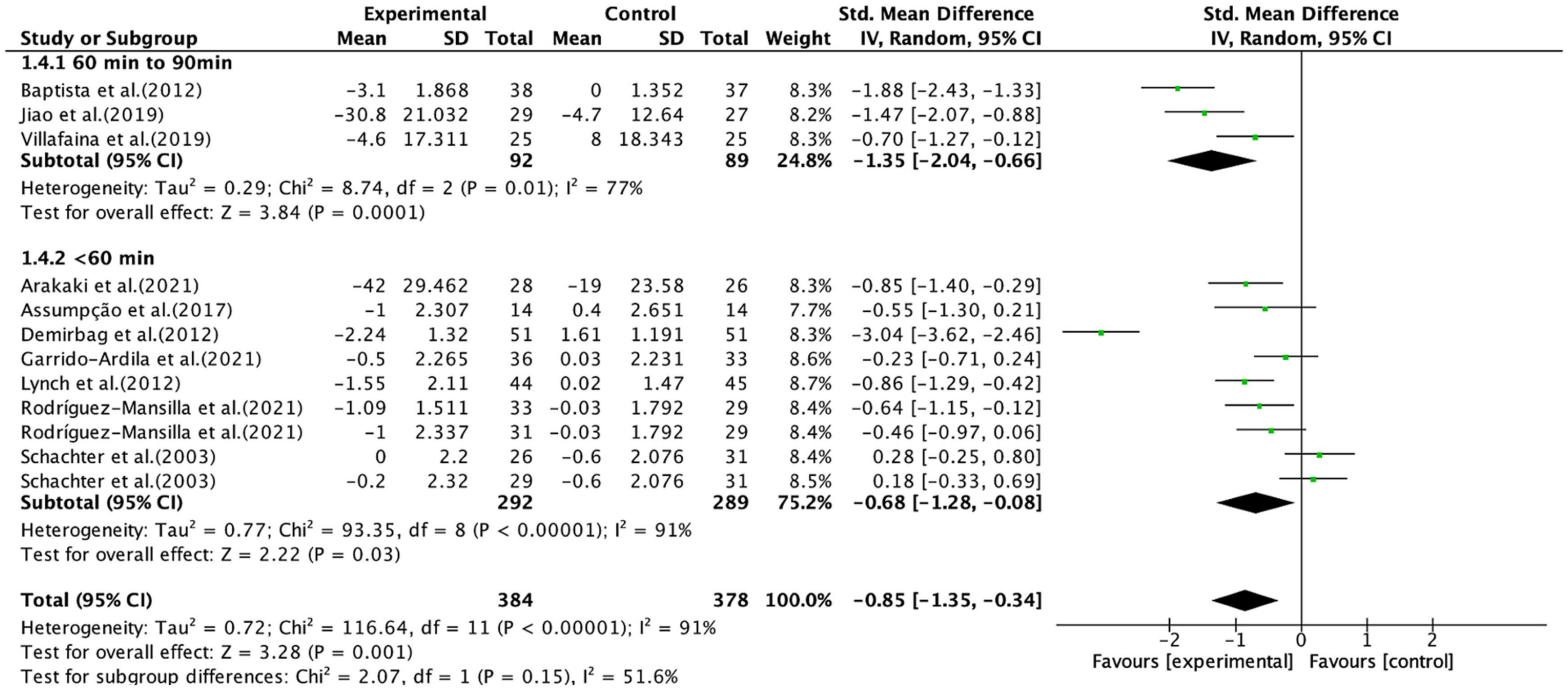
Subgroup analysis by duration of aerobic exercise per session (<60 min, 60–90 min) on pain outcomes.

In addition, aerobic exercise lasting <60 min per session (SMD, −0.68; 95% CI, −1.28 to −0.08; *p* = 0.03; *I*^2^ = 91%) and 60–90 min per session (SMD, −1.35; 95% CI, −2.04 to −0.66; *p* = 0.0001; *I*^2^ = 77%, Figure 7) significantly alleviated pain, with longer session durations showing better efficacy.

**Figure 8:**
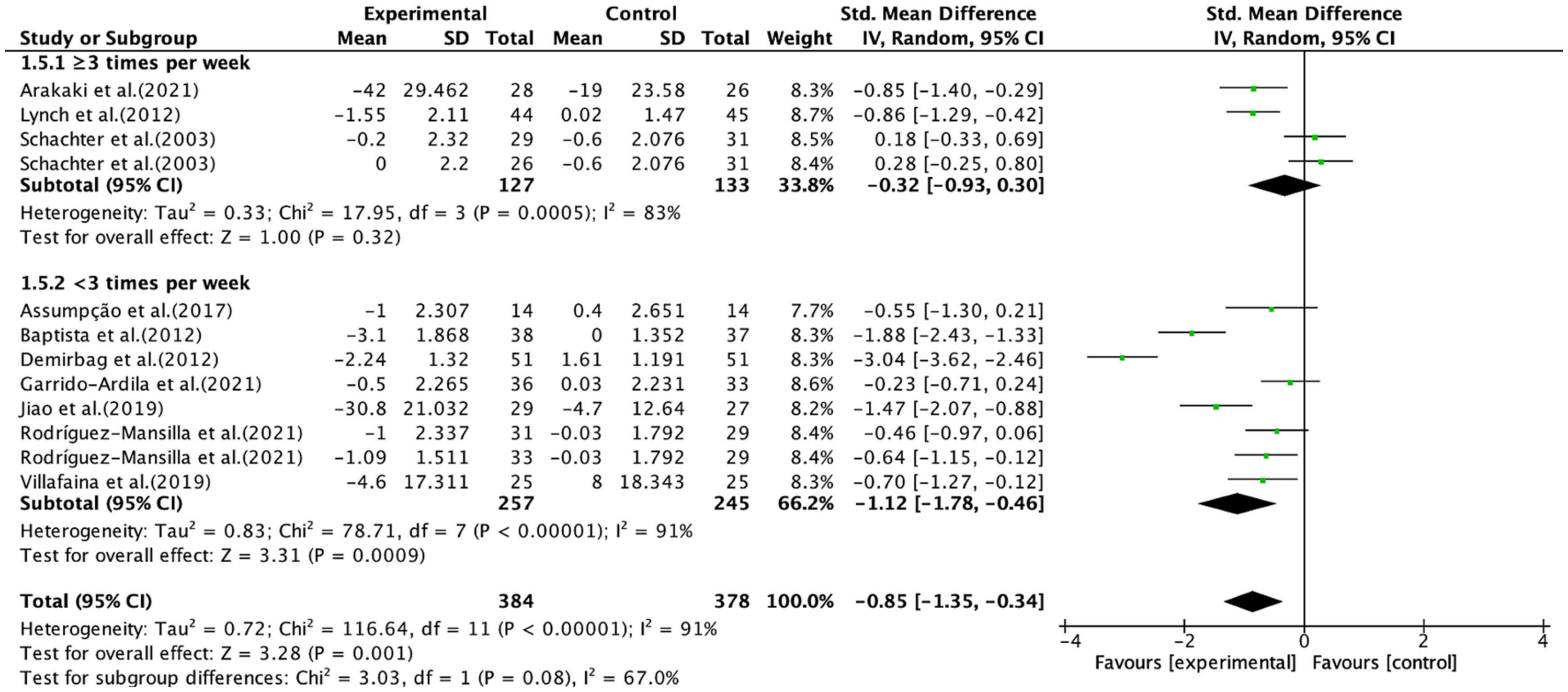
Subgroup analysis by frequency of aerobic exercise (<3 times, ≥3 times) on pain outcomes.

Furthermore, aerobic exercise conducted for <3 times per week significantly alleviated pain (SMD, −1.12; 95% CI, −1.78 to −0.46; *p* = 0.0009; *I*^2^ = 91%), while aerobic exercise conducted for ≥3 times per week had no significant effect on pain (SMD, −0.32; 95% CI, −0.93 to −0.30; *p* = 0.32; *I*^2^ = 83%, Figure 8).

**Figure 9:**
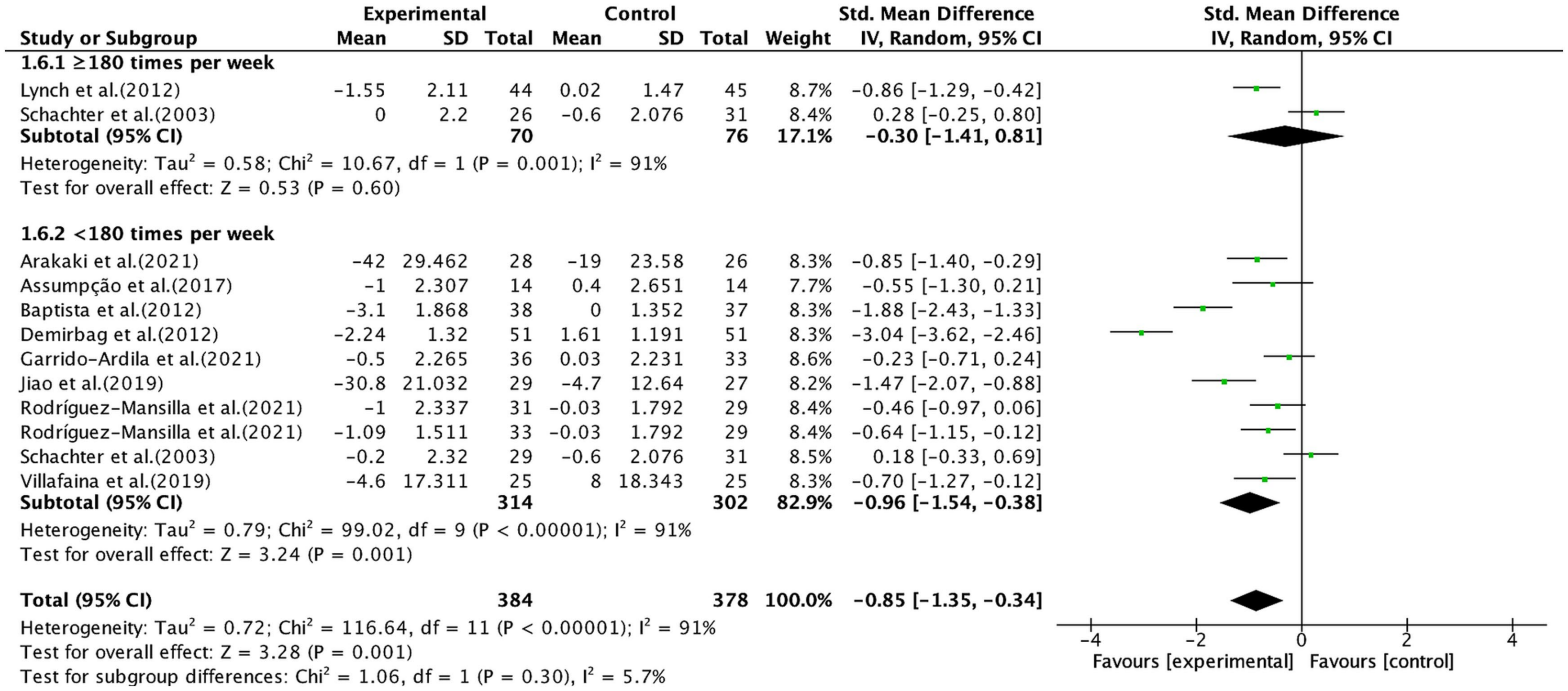
Subgroup analysis by duration of aerobic exercise per week (<180 min, ≥180 min) on pain outcomes.

Finally, aerobic exercise conducted for <180 min per week significantly alleviated pain (SMD, −0.96; 95% CI, −1.54 to −0.38; *p* = 0.001; *I*^2^ = 91%), while aerobic exercise conducted for ≥ 180 min per week showed no significant effect (SMD, −0.30; 95% CI, −1.41 to 0.81; *p* = 0.6; *I*^2^ = 91%, Figure 9).

### Risk of bias

The risk of bias for the included trials was evaluated using the RoB tool, which examines potential sources of bias related to selection, performance, detection, attrition, reporting, and other biases. As illustrated in Supplementary Figure S1, the studies were classified into three categories of overall quality: low, moderate, and high.

According to the GRADE framework, the certainty of evidence ranged from very low to moderate across outcomes (Supplementary Table S3). Specifically, evidence certainty was rated as very low for pain and fatigue outcomes, primarily due to serious performance bias, substantial heterogeneity, and evidence of reporting bias indicated by funnel plot asymmetry. In contrast, the certainty of evidence for QOL was rated as moderate, with downgrading mainly driven by performance bias, while inconsistency, indirectness, and imprecision were not considered serious.

### Publication bias

The funnel plot for pain exhibits a marked right-skewed distribution, with data points densely concentrated on the left and sparsely distributed on the right (Supplementary Figure S2). The funnel plot for fatigue revealed clear asymmetry and a relatively small sample size, suggesting potential small-study effects (Supplementary Figure S3). In contrast, the funnel plot for QOL appeared approximately symmetrical, with data points evenly distributed within the confidence intervals (Supplementary Figure S4). However, Egger’s test results indicated that studies with small sample sizes did not significantly impact pain (*p* = 0.062), fatigue (*p* = 0.588), and QOL (*p* = 0.859) outcomes.

### Sensitivity analysis

Sensitivity analysis demonstrated that the positive effects of exercise on pain (Supplementary Figure S5), fatigue (Supplementary Figure S6), and QOL (Supplementary Figure S7) in fibromyalgia patients remained stable in both direction and magnitude, irrespective of individual study exclusions.

## Discussion

### Main findings

This study examined the effect of exercise on pain, fatigue, and QOL in fibromyalgia patients, with the additional aim of determining the optimal exercise prescription for this population. Across the 24 studies included, exercise were consistently associated with reductions in pain and fatigue as well as improvements in QOL in fibromyalgia patients. Subgroup analyses indicated that aerobic exercise performed for at least 8 weeks, fewer than 3 times per week, with each session lasting 60–90 min and a total weekly duration of less than 180 min, was most effective in alleviating pain.

### Effects of exercise on pain in fibromyalgia patients

Our findings indicated that exercise was associated with significant reductions in pain in fibromyalgia patients, aligning with previous studies (19, 31, 33, 47, 59, 60). Interventions, including aerobic exercises such as walking and swimming, resistance exercises, home-based exercise programs, and exergaming, all positively contributed to pain relief.

Central sensitization, a key mechanism in fibromyalgia, is characterized by hyperalgesia and allodynia (61). Neurotransmitter imbalances, such as reduced levels of norepinephrine, serotonin, and dopamine (62), and elevated levels of substance P and glutamate (63, 64), contribute to impaired pain inhibition. Hypothalamic–pituitary–adrenal (HPA) axis dysfunction can exacerbate neuroinflammation and pain (65, 66), and is linked to emotional disturbances like anxiety, fatigue, and insomnia, factors that may act synergistically to amplify pain perceptions, potentially creating a cyclical relationship (67, 68).

Exercise may modulate the endogenous analgesic system by potentially activating descending inhibitory pathways and modulating neurotransmitters such as opioid peptides, brain-derived neurotrophic factor (BDNF), 5-hydroxytryptamine (5-HT), and gamma-aminobutyric acid (GABA) (12, 69, 70). It may also be associated with increased pain thresholds, reduced pain sensitivity (71), stabilized HPA axis function (72), and improved physical function and pain management through diverse modalities like endurance and resistance exercises (28, 73–75). These potential mechanistic links are supported by preclinical and observational data, though causal relationships cannot be confirmed by the meta-analytic design of the present study.

However, our findings exhibit some discrepancies with previous studies in certain aspects, with these differences likely attributable to methodological and analytical variations across investigations. While previous studies (31, 34) excluded exercises like Tai Chi and Yoga, one study (33) showed that technique-based exercises effectively alleviated pain and anxiety in fibromyalgia patients. We included these exercises to capture a more comprehensive range of exercise modalities used in clinical practice, which may explain the divergent findings related to intervention type. Additionally, one included study (27) reported a negative association between exercise and pain outcomes, which we hypothesize may be linked to potential overtraining in the study’s participant cohort—an issue that highlights the critical role of exercise dosage in fibromyalgia interventions. To further explore this relationship, we conducted a subgroup analysis on training dosage to explore the optimal exercise prescription. Finally, our meta-analysis included a larger number of studies and attempted to validate observed associations through subgroup and sensitivity analyses, which may account for more nuanced findings compared to smaller, single-arm investigations.

Substantial heterogeneity was observed across pain-related outcomes. Subgroup analyses indicated particularly high heterogeneity within the aerobic exercise subgroup (*I*^2^ = 89%), followed by multicomponent training (*I*^2^ = 78%), whereas resistance exercise demonstrated negligible heterogeneity (*I*^2^ = 0%). This pronounced variability is likely attributable to multiple factors. First, considerable differences in baseline pain severity across study populations may have influenced the magnitude of pain reduction and intervention responsiveness. Second, heterogeneity may have been amplified by the use of diverse pain assessment instruments, which vary in sensitivity, construct focus, and scoring methods. In addition, substantial variability exists in aerobic exercise protocols, particularly with respect to intensity, duration, and progression strategies (44, 47), and this may engage distinct pain modulation pathways, further contributing to between-study inconsistency.

### Effects of various exercise moderators on pain in fibromyalgia patients

To determine the optimal exercise prescription, we conducted subgroup analyses based on intervention type, duration, session duration, frequency, and weekly time.

Subgroup analysis by intervention type showed that aerobic exercise, resistance exercise, and multicomponent training were all associated with pain reduction, with aerobic exercise being most effective. This aligns with the results of Bircan et al. (76), which showed that aerobic exercise more significantly alleviated pain in fibromyalgia patients compared to resistance exercise. For fibromyalgia patients, the American Pain Society and the guidelines of the Association of the Scientific Medical Societies in Germany gave the highest grade of recommendation for aerobic exercise (77). Furthermore, aerobic exercise is suitable for beginners with no exercise experience, individuals with chronic conditions, or elderly people with mobility issues (78). Aerobic exercise can include low-intensity, rhythmic, and diverse exercise modalities, which have significant benefits for cardiovascular health, metabolic function, and other areas (77, 79). Therefore, we further conducted a subgroup analysis on aerobic exercise to identify potential optimal prescription parameters for this widely recommended modality.

In a subgroup analysis of intervention duration, it was observed that ≥8 weeks of aerobic exercise was associated with significant pain reduction, while <8 weeks of aerobic exercise showed no significant effect in fibromyalgia patients, consistent with previous studies. Albuquerque et al. (32) suggested that at least 13 weeks of aerobic exercise is most effective for alleviating pain in fibromyalgia patients, highlighting the potential dose–response relationship between exercise duration and pain outcomes. Fibromyalgia is not caused by acute inflammation or a single injury; rather, it results from long-term neurotransmitter imbalances, dysfunction of descending pain inhibition pathways, HPA axis dysregulation, and other factors. It is a chronic musculoskeletal pain condition, not acute pain (80, 81), and thus short-term exercise may be insufficient to induce meaningful changes in these long-standing physiological alterations. This mechanistic rationale supports the observed association between longer exercise durations and pain reduction, though causal inferences are not possible.

Subgroup analysis of session duration showed that both <60 min and 60–90 min of aerobic exercise were associated with significant pain reduction, with the 60–90 min duration demonstrating superior effects. It was found that pain thresholds increased when reaching 75% of the maximal oxygen uptake (VO_2_max) during aerobic exercise (82). Since exercise duration and intensity influence oxygen consumption, a session length of 60–90 min may be closer to the threshold required to elevate pain tolerance. Busch et al. (83) recommended that aerobic exercise should begin at an intensity below the individual’s physical capacity and gradually progress to moderate intensity to avoid symptom exacerbation, a clinical consideration that aligns with our observation of superior effects with 60–90 min sessions, as longer moderate-intensity sessions may balance physiological benefit and tolerability. Therefore, we consider a session duration of 60–90 min a potentially feasible and effective parameter for aerobic exercise prescriptions in this population.

In terms of frequency, our results showed that aerobic exercise performed less than three times per week was associated with significant pain reduction, whereas interventions with a frequency of three or more sessions per week did not yield significant effects. This is consistent with a previous study (77). Furthermore, one included study (27) reported that exercising 3–5 times per week actually exacerbated pain in fibromyalgia patients. Compared to healthy individuals, fibromyalgia patients exhibit lower pain thresholds and tolerance, as well as heightened pain sensitivity and abnormal pain perception (1), making them less capable of tolerating high-intensity exercise (10). Additionally, pain can lead to emotional distress and muscle weakness, which in turn may trigger exercise-related anxiety, reduce voluntary activity and muscle tone, and ultimately worsen both pain and fatigue (84). Therefore, a high exercise frequency may contribute to increased fatigue and reduced exercise adherence, potentially leading to the absence of an observed pain reduction effect. Similarly, our results indicated that a total weekly aerobic exercise duration of less than 180 min was associated with significant pain reduction, whereas durations equal to or greater than 180 min had no significant effect, which is consistent with previous studies (33, 85). Taken together, these findings suggest that excessively long session durations or high exercise frequencies may be associated with worse symptom outcomes in fibromyalgia patients, potentially due to reduced tolerability and adherence. Thus, we propose an aerobic exercise prescription characterized by “moderate session duration and reduced frequency,” specifically 60–90 min per session, twice per week, ensuring that the total weekly exercise time does not exceed 180 min, as a potentially optimal exploratory parameter set for future interventional testing.

### Effects of exercise on fatigue in fibromyalgia patients

Our study confirmed that exercise was associated with significant improvements in fatigue in fibromyalgia patients, consistent with previous studies. Estévez-López et al. (86) reported exercise reduces fatigue sensation in fibromyalgia patients. Chronic pain in fibromyalgia is accompanied by fatigue and sleep disturbances (87), and these symptoms are likely bidirectionally associated, with each exacerbating the other. Fatigue is closely intertwined with pain, sleep disorders, and cognitive dysfunction in fibromyalgia, creating a complex symptom cluster that may be modifiable by non-pharmacological interventions like exercise. Wu et al. (88) found exercise-based gaming interventions improved pain and fatigue by enhancing muscular strength and reducing perceived fatigue. Similarly, Bidonde et al. (89) showed multicomponent training simultaneously improved pain and fatigue. Exercise may also alleviate fatigue by modulating the HPA axis (90), one potential mechanistic pathway connecting exercise to fatigue reduction in this population. Therefore, we proposed a potential bidirectional associative relationship between pain and fatigue in fibromyalgia: pain reduction may be associated with reduced fatigue, and fatigue reduction may in turn be linked to increased physical activity participation, ultimately potentially mitigating the negative impact of fibromyalgia symptoms.

### Effects of exercise on QOL in fibromyalgia patients

This study demonstrated that exercise was associated with significant improvements in QOL in fibromyalgia patients. Rodríguez-Almagro et al. (33) found that exercise improved the physical and psychological status of fibromyalgia patients by reducing pain, fatigue, and anxiety, thereby establishing exercise as an excellent therapy for improving QOL. Kim et al. (91) reported that flexibility exercises alleviated muscle tension and improved QOL by reducing pain, fatigue, and sleep disturbances. Similarly, Lazaridou et al. (92) discovered that Yoga positively alleviated pain, anxiety, and other symptoms in fibromyalgia patients, enhancing personal functioning and QOL. In addition, it has been shown that pain reduction also alleviates psychological fear, increasing exercise acceptance and further mitigating fibromyalgia’s impact (93). Therefore, we conclude that exercise, pain, fatigue, and QOL are interconnected in fibromyalgia, with observational data suggesting that: exercise may be associated with alleviated pain and fatigue, and the relief of these core symptoms may act synergistically to promote improvements in QOL in this patient population.

### Limitations

This study has several limitations. First, the included studies employed heterogeneous exercise protocols, encompassing different supervision modes (self-supervised vs. professionally supervised) and training environments (home-based vs. outdoor settings), which may have contributed to variability in intervention effects. For example, professionally supervised programs may yield greater benefits, whereas adherence variability in home-based training could introduce additional heterogeneity. Second, the use of relaxation training as a control condition may have attenuated the observed between-group effects, given its established efficacy in improving psychological outcomes. Third, the relatively small number of studies reporting fatigue outcomes may limit the robustness of conclusions for this endpoint. Importantly, most included studies did not adequately report exercise intensity, precluding intensity-based subgroup analyses and constraining interpretation of frequency- and duration-related patterns. In addition, some studies exhibited methodological limitations or incomplete reporting. Future high-quality RCTs should provide standardized and transparent reporting of exercise intensity to support the development of more precise and evidence-informed exercise prescriptions.

## Conclusion

Exercise was associated with improvements in pain, fatigue, and QOL in fibromyalgia patients. Observational patterns from the analyses suggest a potential positive interrelationship among these factors, whereby exercise may mitigate key fibromyalgia-related symptoms by alleviating pain, which in turn may reduce fatigue and contribute to enhanced QOL in this patient population. For clinical consideration, engaging in aerobic exercise for a minimum of 8 weeks (60–90 min per session, twice weekly, with a weekly total of no more than 180 min) may represent a feasible approach to address pain in fibromyalgia patients, though these exercise parameters are exploratory and require further validation. Future research should prioritize longitudinal and interventional studies to confirm the observed associations between exercise and fibromyalgia-related symptoms, and to develop optimized, personalized exercise protocols tailored to the unique clinical needs of fibromyalgia patients.

## Data Availability

The original contributions presented in the study are included in the article/Supplementary material, further inquiries can be directed to the corresponding authors.
