# Supplementary material for: Effects of exercise on pain, fatigue, and quality of life in people with fibromyalgia: a systematic review and meta-analysis of randomized controlled trials
Source: Front Med (Lausanne). 2026 Feb 18;13:1782714. doi: 10.3389/fmed.2026.1782714 (PMC12956681; doi:10.3389/fmed.2026.1782714)
Supplement: Supplementary file 1 [file Data_Sheet_1.pdf]

Supplemental Material

Effects of exercise on pain, fatigue, and quality of life in people with fibromyalgia: a systematic review and meta-analysis of randomized controlled trials

Figure S1. Results of Cochrane risk of bias tool 2

Figure S2. Funnel plot for pain publication bias 3

Figure S3. Funnel plot for fatigue publication bias 4

Figure S4. Funnel plot for quality of life publication bias 5

Figure S5. Sensitivity analysis for pain stability 6

Figure S6. Sensitivity analysis for fatigue stability 7

Figure S7. Sensitivity analysis for quality of life stability 8

Table S1. Search strategies 9

Table S2. Characteristics of the studies included in this meta-analysis 23

Table S3. GRADE summary of evidence 30

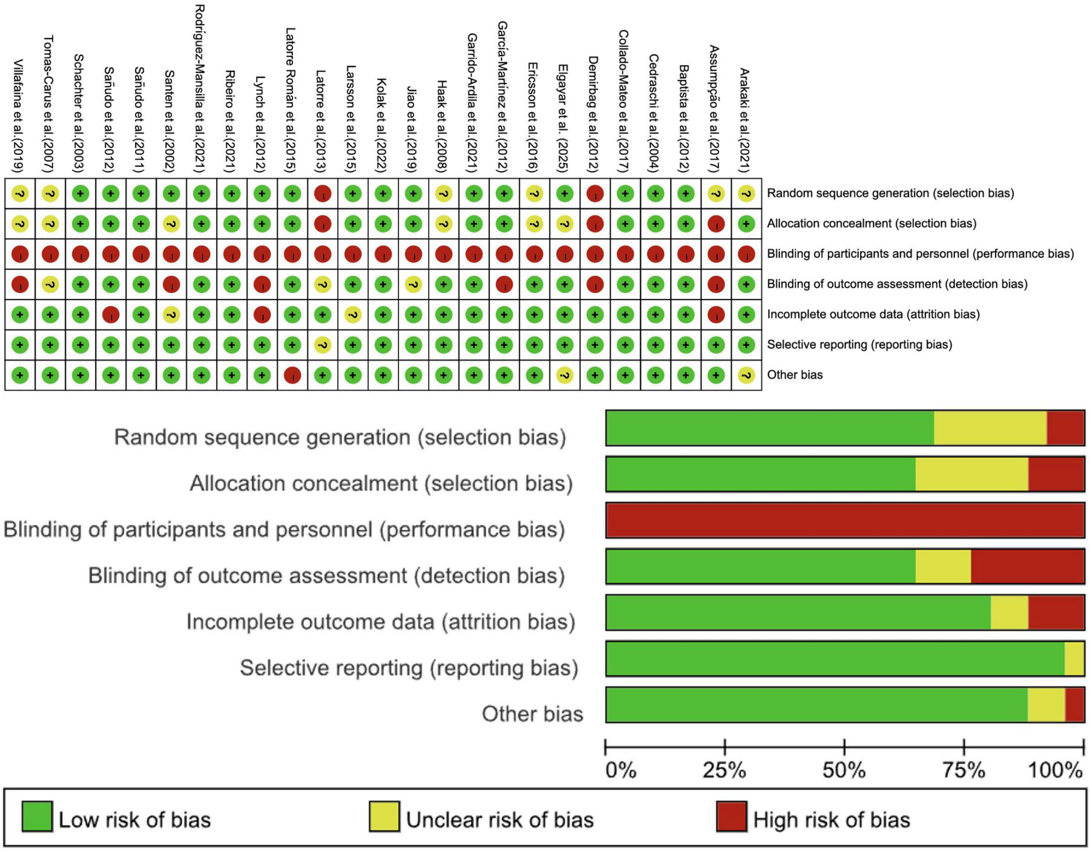

Figure S1. Results of Cochrane risk of bias tool.

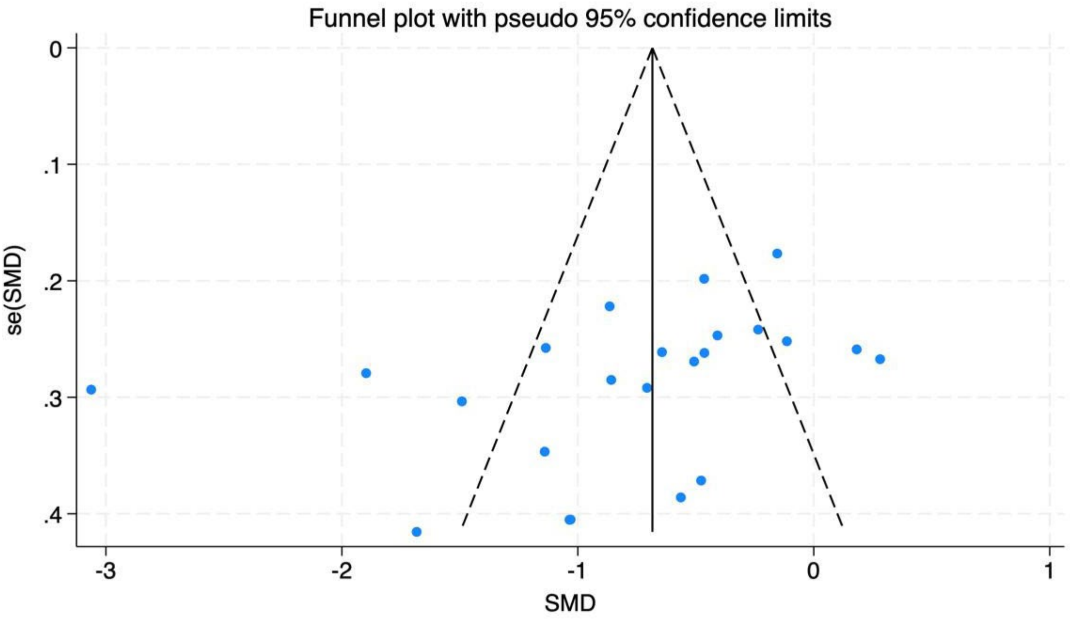

Figure S2. Funnel plot for pain publication bias.

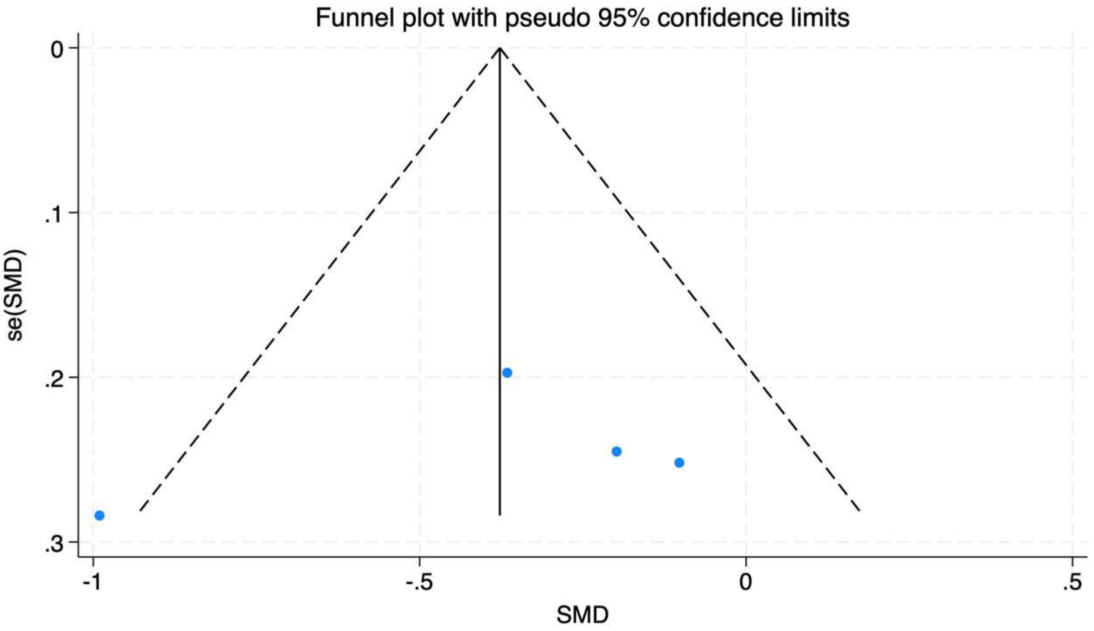

Figure S3. Funnel plot for fatigue publication bias.

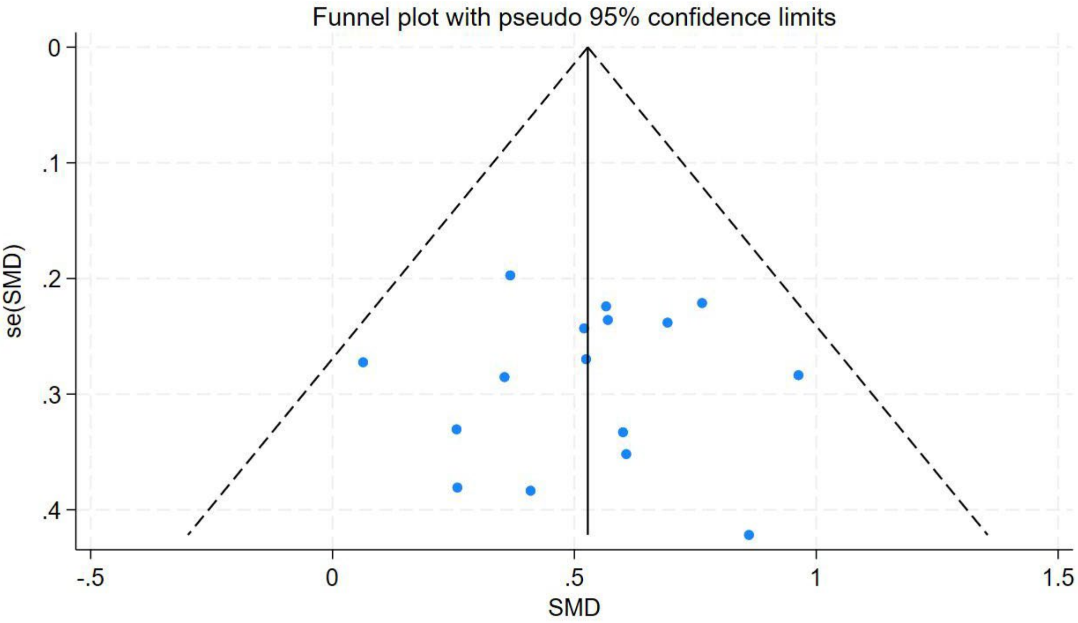

Figure S4. Funnel plot for quality of life publication bias.

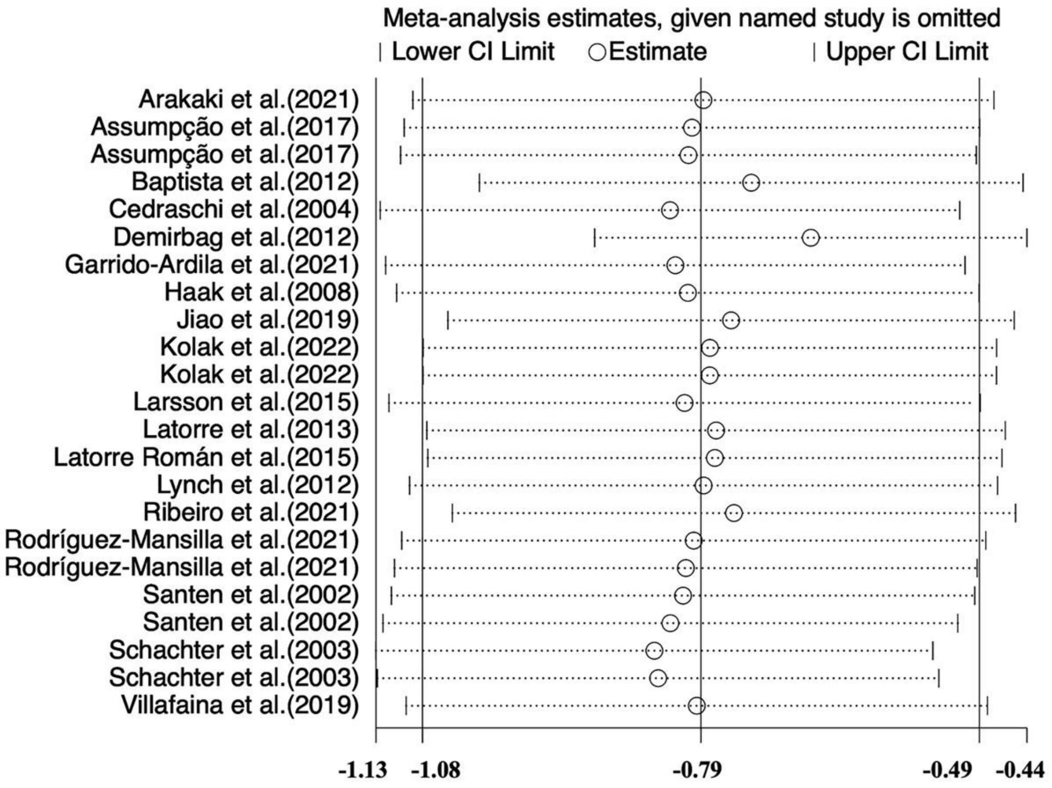

Figure S5. Sensitivity analysis for pain stability.

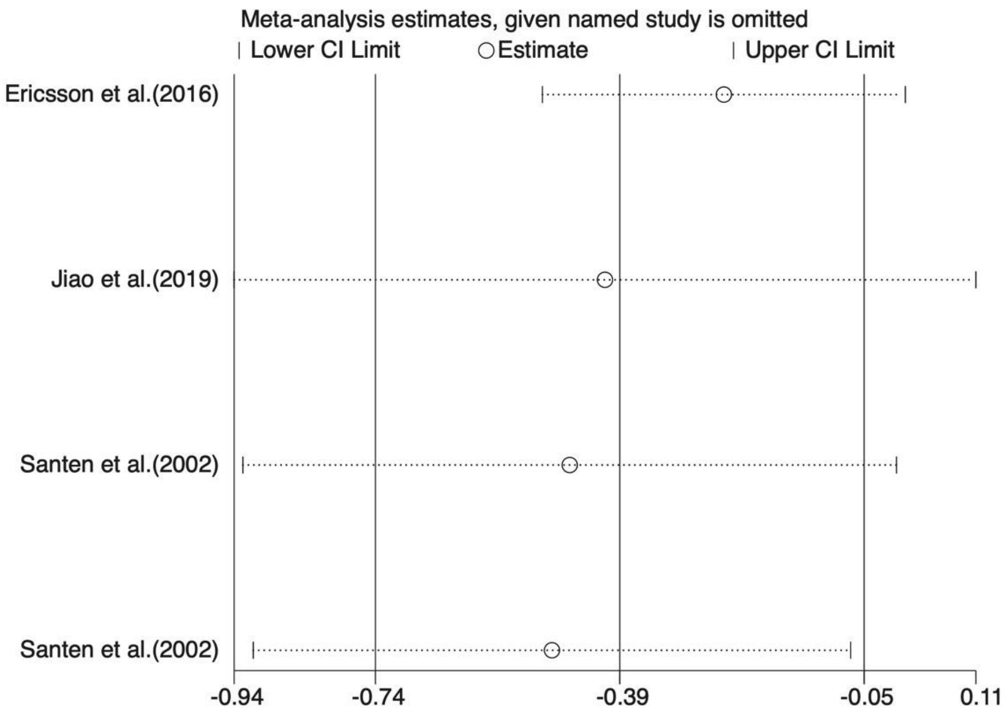

Figure S6. Sensitivity analysis for fatigue stability.

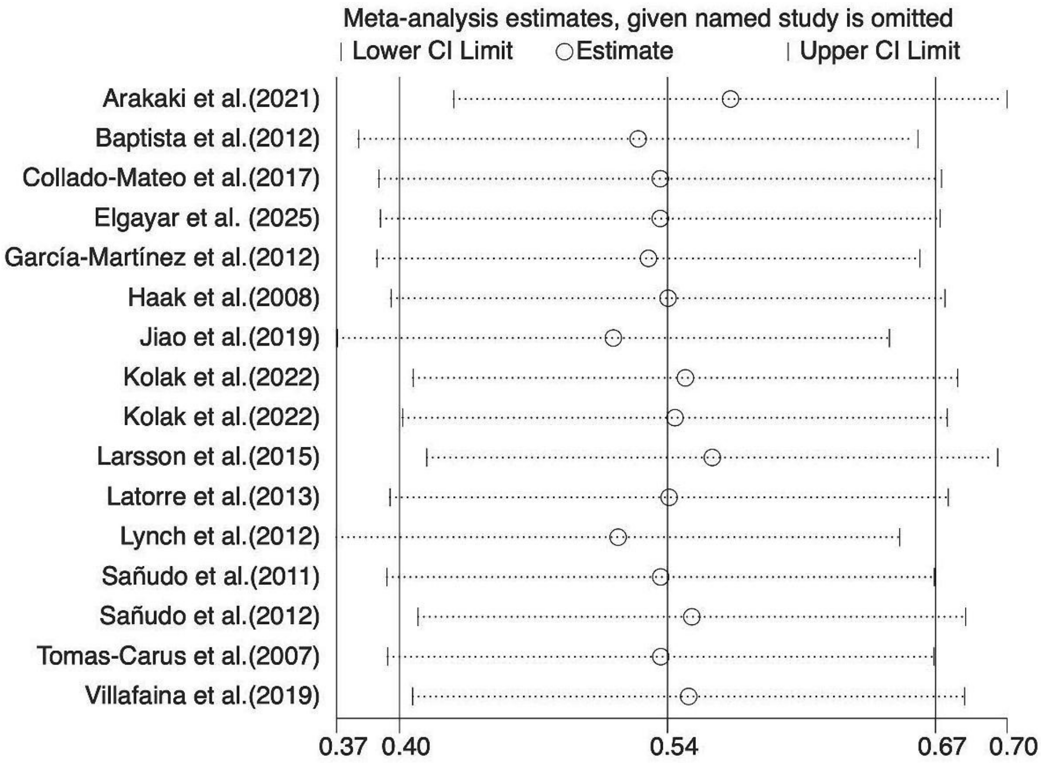

Figure S7. Sensitivity analysis for quality of life stability.

Table S1. Search strategies.

Web of Science

Search Component	Search Number	Search Query
Fibromyalgia Terms	#1	TS= ("Fibromyalgia" OR "Fibromyalgias" OR "Fibromyalgia-Fibromyositis Syndrome" OR "Fibromyalgia Fibromyositis Syndrome" OR "Fibromyalgia-Fibromyositis Syndromes" OR "Syndrome, Fibromyalgia-Fibromyositis" OR "Syndromes, Fibromyalgia-Fibromyositis" OR "Rheumatism, Muscular" OR "Muscular Rheumatism" OR "Fibrositis" OR "Fibrositides" OR "Myofascial Pain Syndrome, Diffuse" OR "Diffuse Myofascial Pain Syndrome" OR "Fibromyositis-Fibromyalgia Syndrome" OR "Fibromyositis Fibromyalgia Syndrome" OR "Fibromyositis-Fibromyalgia Syndromes" OR "Syndrome, Fibromyositis-Fibromyalgia" OR "Syndromes, Fibromyositis-Fibromyalgia" OR "Fibromyalgia, Secondary" OR "Fibromyalgias, Secondary" OR "Secondary Fibromyalgia" OR "Secondary Fibromyalgias" OR "Fibromyalgia, Primary" OR "Fibromyalgias, Primary" OR "Primary Fibromyalgia" OR "Primary Fibromyalgias")
Exercise Terms	#2	TS= ("Exercise" OR "Exercises" OR "Physical exercise programs" OR "Physical Therapy Modalities" OR "Modalities, Physical Therapy" OR "Modality, Physical Therapy" OR "Physical Therapy Modality" OR "Physiotherapy" OR "Physiotherapies" OR "Physical Therapy Techniques" OR "Physical Therapy Technique" OR "Techniques, Physical Therapy" OR "Exercise

		Movement Techniques" OR "Movement Techniques, Exercise" OR "Exercise Movement Technics" OR "Exercise Therapy" OR "Therapy, Exercise" OR "Exercise Therapies" OR "Therapies, Exercise" OR "Exercise, Physical" OR "Exercises, Physical" OR "Physical Exercise" OR "Physical Exercises" OR "Exercise, Isometric" OR "Exercises, Isometric" OR "Isometric Exercises" OR "Isometric Exercise" OR "Exercise, Aerobic" OR "Aerobic Exercises" OR "Aerobic Exercise" OR "Resistance Training" OR "Training, Resistance" OR "Strength Training" OR "Training, Strength" OR "Weight-Lifting Strengthening Program" OR "Strengthening Program, Weight-Lifting" OR "Strengthening Programs, Weight-Lifting" OR "Weight Lifting Strengthening Program" OR "Weight-Lifting Strengthening Programs" OR "Weight-Lifting Exercise Program" OR "Exercise Program, Weight-Lifting" OR "Exercise Programs, Weight-Lifting" OR "Weight Lifting Exercise Program" OR "Weight-Lifting Exercise Programs" OR "Weight-Bearing Strengthening Program" OR "Strengthening Program, Weight-Bearing" OR "Strengthening Programs, Weight-Bearing" OR "Weight Bearing Strengthening Program" OR "Weight-Bearing Strengthening Programs" OR "Weight-Bearing Exercise Program" OR "Exercise Program, Weight-Bearing" OR "Exercise Programs, Weight-Bearing" OR "Weight Bearing Exercise Program" OR "Weight-Bearing Exercise Programs")
Pain Terms	#3	TS= ("Pain" OR "Pain, Burning" OR "Burning Pain" OR

		"Burning Pains" OR "Pains, Burning" OR "Suffering, Physical" OR "Physical Suffering" OR "Physical Sufferings" OR "Sufferings, Physical" OR "Pain, Migratory" OR "Migratory Pain" OR "Migratory Pains" OR "Pains, Migratory" OR "Pain, Radiating" OR "Pains, Radiating" OR "Radiating Pain" OR "Radiating Pains" OR "Pain, Splitting" OR "Pains, Splitting" OR "Splitting Pain" OR "Splitting Pains" OR "Ache" OR "Aches" OR "Pain, Crushing" OR "Crushing Pain" OR "Crushing Pains" OR "Pains, Crushing")
Fatigue Terms	#4	TS= ("Fatigue" OR "Lassitude" OR "tiredness")
Quality of Life Terms	#5	TS= ("Quality of Life" OR "Life Quality" OR "Health-Related Quality Of Life" OR "Health Related Quality Of Life" OR "HRQOL")
Combined Search	#6	#1 AND #2 AND (#3 OR #4 OR #5)

PubMed

Search Component	Search Number	Search Query
Fibromyalgia Terms	#1	("Fibromyalgia"[Mesh] OR "Fibromyalgia"[tiab] OR "Fibromyalgias"[tiab] OR "Fibromyalgia-Fibromyositis Syndrome"[tiab] OR "Fibromyalgia Fibromyositis Syndrome"[tiab] OR "Fibromyalgia-Fibromyositis Syndromes"[tiab] OR "Syndrome, Fibromyalgia-Fibromyositis"[tiab] OR "Syndromes, Fibromyalgia-Fibromyositis"[tiab] OR "Rheumatism, Muscular"[tiab] OR "Muscular Rheumatism"[tiab] OR

		"Fibrositis"[tiab] OR "Fibrositides"[tiab] OR "Myofascial Pain Syndrome, Diffuse"[tiab] OR "Diffuse Myofascial Pain Syndrome"[tiab] OR "Fibromyositis-Fibromyalgia Syndrome"[tiab] OR "Fibromyositis Fibromyalgia Syndrome"[tiab] OR "Fibromyositis-Fibromyalgia Syndromes"[tiab] OR "Syndrome, Fibromyositis-Fibromyalgia"[tiab] OR "Syndromes, Fibromyositis-Fibromyalgia"[tiab] OR "Fibromyalgia, Secondary"[tiab] OR "Fibromyalgias, Secondary"[tiab] OR "Secondary Fibromyalgia"[tiab] OR "Secondary Fibromyalgias"[tiab] OR "Fibromyalgia, Primary"[tiab] OR "Fibromyalgias, Primary"[tiab] OR "Primary Fibromyalgia"[tiab] OR "Primary Fibromyalgias"[tiab])
Exercise Terms	#2	("Exercise"[Mesh] OR "Exercise"[tiab] OR "Exercises"[tiab] OR "Physical exercise programs"[tiab] OR "Physical Therapy Modalities"[Mesh] OR "Physical Therapy Modalities"[tiab] OR "Modalities, Physical Therapy"[tiab] OR "Modality, Physical Therapy"[tiab] OR "Physical Therapy Modality"[tiab] OR "Physiotherapy"[tiab] OR "Physiotherapies"[tiab] OR "Physical Therapy Techniques"[tiab] OR "Physical Therapy Technique"[tiab] OR "Techniques, Physical Therapy"[tiab] OR "Exercise Movement Techniques"[Mesh] OR "Exercise Movement Techniques"[tiab] OR "Movement Techniques, Exercise"[tiab] OR "Exercise Movement Technics"[tiab] OR "Exercise Therapy"[Mesh] OR "Exercise Therapy"[tiab] OR "Therapy, Exercise"[tiab]

		OR "Exercise Therapies"[tiab] OR "Therapies, Exercise"[tiab] OR "Exercise, Physical"[tiab] OR "Exercises, Physical"[tiab] OR "Physical Exercise"[tiab] OR "Physical Exercises"[tiab] OR "Exercise, Isometric"[tiab] OR "Exercises, Isometric"[tiab] OR "Isometric Exercises"[tiab] OR "Isometric Exercise"[tiab] OR "Exercise, Aerobic"[tiab] OR "Aerobic Exercises"[tiab] OR "Aerobic Exercise"[tiab] OR "Resistance Training"[Mesh] OR "Resistance Training"[tiab] OR "Training, Resistance"[tiab] OR "Strength Training"[tiab] OR "Training, Strength"[tiab] OR "Weight-Lifting Strengthening Program"[tiab] OR "Strengthening Program, Weight-Lifting"[tiab] OR "Strengthening Programs, Weight-Lifting"[tiab] OR "Weight Lifting Strengthening Program"[tiab] OR "Weight-Lifting Strengthening Programs"[tiab] OR "Weight-Lifting Exercise Program"[tiab] OR "Exercise Program, Weight-Lifting"[tiab] OR "Exercise Programs, Weight-Lifting"[tiab] OR "Weight Lifting Exercise Program"[tiab] OR "Weight-Lifting Exercise Programs"[tiab] OR "Weight-Bearing Strengthening Program"[tiab] OR "Strengthening Program, Weight-Bearing"[tiab] OR "Strengthening Programs, Weight-Bearing"[tiab] OR "Weight Bearing Strengthening Program"[tiab] OR "Weight-Bearing Strengthening Programs"[tiab] OR "Weight-Bearing Exercise Program"[tiab] OR "Exercise Program, Weight-Bearing"[tiab] OR "Exercise Programs,

		Weight-Bearing"[tiab] OR "Weight Bearing Exercise Program"[tiab] OR "Weight-Bearing Exercise Programs"[tiab])
Pain Terms	#3	("Pain"[Mesh] OR "Pain"[tiab] OR "Pain, Burning"[tiab] OR "Burning Pain"[tiab] OR "Burning Pains"[tiab] OR "Pains, Burning"[tiab] OR "Suffering, Physical"[tiab] OR "Physical Suffering"[tiab] OR "Physical Sufferings"[tiab] OR "Sufferings, Physical"[tiab] OR "Pain, Migratory"[tiab] OR "Migratory Pain"[tiab] OR "Migratory Pains"[tiab] OR "Pains, Migratory"[tiab] OR "Pain, Radiating"[tiab] OR "Pains, Radiating"[tiab] OR "Radiating Pain"[tiab] OR "Radiating Pains"[tiab] OR "Pain, Splitting"[tiab] OR "Pains, Splitting"[tiab] OR "Splitting Pain"[tiab] OR "Splitting Pains"[tiab] OR "Ache"[tiab] OR "Aches"[tiab] OR "Pain, Crushing"[tiab] OR "Crushing Pain"[tiab] OR "Crushing Pains"[tiab] OR "Pains, Crushing"[tiab])
Fatigue Terms	#4	("Fatigue"[Mesh] OR "Fatigue"[tiab] OR "Lassitude"[tiab] OR "tiredness"[tiab])
Quality of Life Terms	#5	("Quality of Life"[Mesh] OR "Quality of Life"[tiab] OR "Life Quality"[tiab] OR "Health-Related Quality Of Life"[tiab] OR "Health Related Quality Of Life"[tiab] OR "HRQOL"[tiab])
Combined Search	#6	#1 AND #2 AND (#3 OR #4 OR #5)

Cochrane Library

Search	Search	Search Query

Component	Number	
Fibromyalgia Terms	#1	(MeSH descriptor: [Fibromyalgia] explode all trees) OR (fibromyalgia OR fibromyalgias OR "fibromyalgia-fibromyositis syndrome" OR "fibromyalgia fibromyositis syndrome" OR "fibromyalgia-fibromyositis syndromes" OR "syndrome, fibromyalgia-fibromyositis" OR "syndromes, fibromyalgia-fibromyositis" OR "rheumatism, muscular" OR "muscular rheumatism" OR fibrositis OR fibrositides OR "myofascial pain syndrome, diffuse" OR "diffuse myofascial pain syndrome" OR "fibromyositis-fibromyalgia syndrome" OR "fibromyositis fibromyalgia syndrome" OR "fibromyositis-fibromyalgia syndromes" OR "syndrome, fibromyositis-fibromyalgia" OR "syndromes, fibromyositis-fibromyalgia" OR "fibromyalgia, secondary" OR "fibromyalgias, secondary" OR "secondary fibromyalgia" OR "secondary fibromyalgias" OR "fibromyalgia, primary" OR "fibromyalgias, primary" OR "primary fibromyalgia" OR "primary fibromyalgias"):ti,ab,kw
Exercise Terms	#2	(MeSH descriptor: [Exercise] explode all trees) OR (MeSH descriptor: [Exercise Therapy] explode all trees) OR (MeSH descriptor: [Resistance Training] explode all trees) OR (exercise OR exercises OR "physical exercise programs" OR "physical therapy modalities" OR "modalities, physical therapy" OR "modality, physical therapy" OR "physical therapy modality" OR physiotherapy OR physiotherapies OR "physical

		therapy techniques" OR "physical therapy technique" OR "techniques, physical therapy" OR "exercise movement techniques" OR "movement techniques, exercise" OR "exercise movement technics" OR "exercise therapy" OR "therapy, exercise" OR "exercise therapies" OR "therapies, exercise" OR "exercise, physical" OR "exercises, physical" OR "physical exercise" OR "physical exercises" OR "exercise, isometric" OR "exercises, isometric" OR "isometric exercises" OR "isometric exercise" OR "exercise, aerobic" OR "aerobic exercises" OR "aerobic exercise" OR "resistance training" OR "training, resistance" OR "strength training" OR "training, strength" OR "weight-lifting strengthening program" OR "strengthening program, weight-lifting" OR "strengthening programs, weight-lifting" OR "weight lifting strengthening program" OR "weight-lifting strengthening programs" OR "weight-lifting exercise program" OR "exercise program, weight-lifting" OR "exercise programs, weight-lifting" OR "weight lifting exercise program" OR "weight-lifting exercise programs" OR "weight-bearing strengthening program" OR "strengthening program, weight-bearing" OR "strengthening programs, weight-bearing" OR "weight bearing strengthening program" OR "weight-bearing strengthening programs" OR "weight-bearing exercise program" OR "exercise program, weight-bearing" OR "exercise programs, weight-bearing" OR "weight bearing exercise program" OR "weight-bearing exercise

		programs"):ti,ab,kw
Pain Terms	#3	(MeSH descriptor: [Pain] explode all trees) OR (pain OR "pain, burning" OR "burning pain" OR "burning pains" OR "pains, burning" OR "suffering, physical" OR "physical suffering" OR "physical sufferings" OR "sufferings, physical" OR "pain, migratory" OR "migratory pain" OR "migratory pains" OR "pains, migratory" OR "pain, radiating" OR "pains, radiating" OR "radiating pain" OR "radiating pains" OR "pain, splitting" OR "pains, splitting" OR "splitting pain" OR "splitting pains" OR ache OR aches OR "pain, crushing" OR "crushing pain" OR "crushing pains" OR "pains, crushing"):ti,ab,kw
Fatigue Terms	#4	(MeSH descriptor: [Fatigue] explode all trees) OR (fatigue OR lassitude OR tiredness):ti,ab,kw
Quality of Life Terms	#5	(MeSH descriptor: [Quality of Life] explode all trees) OR ("quality of life" OR "life quality" OR "health-related quality of life" OR "health related quality of life" OR hrqol):ti,ab,kw
Combined Search	#6	#1 AND #2 AND (#3 OR #4 OR #5)

Embase

Search Component	Search Number	Search Query
Fibromyalgia Terms	#1	'fibromyalgia'/exp OR 'fibromyalgia':ti,ab,kw OR 'fibromyalgias':ti,ab,kw OR 'fibromyalgia-fibromyositis syndrome':ti,ab,kw OR 'fibromyalgia fibromyositis syndrome':ti,ab,kw OR 'fibromyalgia-fibromyositis

		syndromes':ti,ab,kw OR 'syndrome, fibromyalgia-fibromyositis':ti,ab,kw OR 'syndromes, fibromyalgia-fibromyositis':ti,ab,kw OR 'rheumatism, muscular':ti,ab,kw OR 'muscular rheumatism':ti,ab,kw OR 'fibrositis':ti,ab,kw OR 'fibrositides':ti,ab,kw OR 'myofascial pain syndrome, diffuse':ti,ab,kw OR 'diffuse myofascial pain syndrome':ti,ab,kw OR 'fibromyositis-fibromyalgia syndrome':ti,ab,kw OR 'fibromyositis fibromyalgia syndrome':ti,ab,kw OR 'fibromyositis-fibromyalgia syndromes':ti,ab,kw OR 'syndrome, fibromyositis-fibromyalgia':ti,ab,kw OR 'syndromes, fibromyositis-fibromyalgia':ti,ab,kw OR 'fibromyalgia, secondary':ti,ab,kw OR 'fibromyalgias, secondary':ti,ab,kw OR 'secondary fibromyalgia':ti,ab,kw OR 'secondary fibromyalgias':ti,ab,kw OR 'fibromyalgia, primary':ti,ab,kw OR 'fibromyalgias, primary':ti,ab,kw OR 'primary fibromyalgia':ti,ab,kw OR 'primary fibromyalgias':ti,ab,kw
Exercise Terms	#2	'exercise'/exp OR 'exercise therapy'/exp OR 'resistance training'/exp OR 'exercise':ti,ab,kw OR 'exercises':ti,ab,kw OR 'physical exercise programs':ti,ab,kw OR 'physical therapy modalities':ti,ab,kw OR 'modalities, physical therapy':ti,ab,kw OR 'modality, physical therapy':ti,ab,kw OR 'physical therapy modality':ti,ab,kw OR 'physiotherapy':ti,ab,kw OR 'physiotherapies':ti,ab,kw OR 'physical therapy techniques':ti,ab,kw OR 'physical therapy

		technique':ti,ab,kw OR 'techniques, physical therapy':ti,ab,kw OR 'exercise movement techniques':ti,ab,kw OR 'movement techniques, exercise':ti,ab,kw OR 'exercise movement technics':ti,ab,kw OR 'exercise therapy':ti,ab,kw OR 'therapy, exercise':ti,ab,kw OR 'exercise therapies':ti,ab,kw OR 'therapies, exercise':ti,ab,kw OR 'exercise, physical':ti,ab,kw OR 'exercises, physical':ti,ab,kw OR 'physical exercise':ti,ab,kw OR 'physical exercises':ti,ab,kw OR 'exercise, isometric':ti,ab,kw OR 'exercises, isometric':ti,ab,kw OR 'isometric exercises':ti,ab,kw OR 'isometric exercise':ti,ab,kw OR 'exercise, aerobic':ti,ab,kw OR 'aerobic exercises':ti,ab,kw OR 'aerobic exercise':ti,ab,kw OR 'resistance training':ti,ab,kw OR 'training, resistance':ti,ab,kw OR 'strength training':ti,ab,kw OR 'training, strength':ti,ab,kw OR 'weight-lifting strengthening program':ti,ab,kw OR 'strengthening program, weight-lifting':ti,ab,kw OR 'strengthening programs, weight-lifting':ti,ab,kw OR 'weight lifting strengthening program':ti,ab,kw OR 'weight-lifting strengthening programs':ti,ab,kw OR 'weight-lifting exercise program':ti,ab,kw OR 'exercise program, weight-lifting':ti,ab,kw OR 'exercise programs, weight-lifting':ti,ab,kw OR 'weight lifting exercise program':ti,ab,kw OR 'weight-lifting exercise programs':ti,ab,kw OR 'weight-bearing strengthening program':ti,ab,kw OR 'strengthening program, weight-bearing':ti,ab,kw OR 'strengthening programs,

		weight-bearing':ti,ab,kw OR 'weight bearing strengthening program':ti,ab,kw OR 'weight-bearing strengthening programs':ti,ab,kw OR 'weight-bearing exercise program':ti,ab,kw OR 'exercise program, weight-bearing':ti,ab,kw OR 'exercise programs, weight-bearing':ti,ab,kw OR 'weight bearing exercise program':ti,ab,kw OR 'weight-bearing exercise programs':ti,ab,kw
Pain Terms	#3	'pain'/exp OR 'pain':ti,ab,kw OR 'pain, burning':ti,ab,kw OR 'burning pain':ti,ab,kw OR 'burning pains':ti,ab,kw OR 'pains, burning':ti,ab,kw OR 'suffering, physical':ti,ab,kw OR 'physical suffering':ti,ab,kw OR 'physical sufferings':ti,ab,kw OR 'sufferings, physical':ti,ab,kw OR 'pain, migratory':ti,ab,kw OR 'migratory pain':ti,ab,kw OR 'migratory pains':ti,ab,kw OR 'pains, migratory':ti,ab,kw OR 'pain, radiating':ti,ab,kw OR 'pains, radiating':ti,ab,kw OR 'radiating pain':ti,ab,kw OR 'radiating pains':ti,ab,kw OR 'pain, splitting':ti,ab,kw OR 'pains, splitting':ti,ab,kw OR 'splitting pain':ti,ab,kw OR 'splitting pains':ti,ab,kw OR 'ache':ti,ab,kw OR 'aches':ti,ab,kw OR 'pain, crushing':ti,ab,kw OR 'crushing pain':ti,ab,kw OR 'crushing pains':ti,ab,kw OR 'pains, crushing':ti,ab,kw
Fatigue Terms	#4	'fatigue'/exp OR 'fatigue':ti,ab,kw OR 'lassitude':ti,ab,kw OR 'tiredness':ti,ab,kw
Quality of Life Terms	#5	'quality of life'/exp OR 'quality of life':ti,ab,kw OR 'life quality':ti,ab,kw OR 'health-related quality of life':ti,ab,kw OR 'health related quality of life':ti,ab,kw OR 'hrqol':ti,ab,kw

Combined Search	#6	#1 AND #2 AND (#3 OR #4 OR #5)

Scopus

Search Component	Search Number	Search Query
Fibromyalgia Terms	#1	TITLE-ABS-KEY("fibromyalgia" OR "fibromyalgias" OR "fibromyalgia-fibromyositis syndrome" OR "fibromyalgia fibromyositis syndrome" OR "fibromyalgia-fibromyositis syndromes" OR "syndrome, fibromyalgia-fibromyositis" OR "syndromes, fibromyalgia-fibromyositis" OR "rheumatism, muscular" OR "muscular rheumatism" OR "fibrositis" OR "fibrositides" OR "myofascial pain syndrome, diffuse" OR "diffuse myofascial pain syndrome" OR "fibromyositis-fibromyalgia syndrome" OR "fibromyositis fibromyalgia syndrome" OR "fibromyositis-fibromyalgia syndromes" OR "syndrome, fibromyositis-fibromyalgia" OR "syndromes, fibromyositis-fibromyalgia" OR "fibromyalgia, secondary" OR "fibromyalgias, secondary" OR "secondary fibromyalgia" OR "secondary fibromyalgias" OR "fibromyalgia, primary" OR "fibromyalgias, primary" OR "primary fibromyalgia" OR "primary fibromyalgias")
Exercise Terms	#2	TITLE-ABS-KEY("exercise" OR "exercises" OR "physical exercise programs" OR "physical therapy modalities" OR "modalities, physical therapy" OR "modality, physical therapy" OR "physical therapy

		modality" OR "physiotherapy" OR "physiotherapies" OR "physical therapy techniques" OR "physical therapy technique" OR "techniques, physical therapy" OR "exercise movement techniques" OR "movement techniques, exercise" OR "exercise movement technics" OR "exercise therapy" OR "therapy, exercise" OR "exercise therapies" OR "therapies, exercise" OR "exercise, physical" OR "exercises, physical" OR "physical exercise" OR "physical exercises" OR "exercise, isometric" OR "exercises, isometric" OR "isometric exercises" OR "isometric exercise" OR "exercise, aerobic" OR "aerobic exercises" OR "aerobic exercise" OR "resistance training" OR "training, resistance" OR "strength training" OR "training, strength" OR "weight-lifting strengthening program" OR "strengthening program, weight-lifting" OR "strengthening programs, weight-lifting" OR "weight lifting strengthening program" OR "weight-lifting strengthening programs" OR "weight-lifting exercise program" OR "exercise program, weight-lifting" OR "exercise programs, weight-lifting" OR "weight lifting exercise program" OR "weight-lifting exercise programs" OR "weight-bearing strengthening program" OR "strengthening program, weight-bearing" OR "strengthening programs, weight-bearing" OR "weight bearing strengthening program" OR "weight-bearing strengthening programs" OR "weight-bearing exercise program" OR "exercise program, weight-bearing" OR "exercise programs, weight-bearing" OR "weight

		bearing exercise program" OR "weight-bearing exercise programs")
Pain Terms	#3	TITLE-ABS-KEY("pain" OR "pain, burning" OR "burning pain" OR "burning pains" OR "pains, burning" OR "suffering, physical" OR "physical suffering" OR "physical sufferings" OR "sufferings, physical" OR "pain, migratory" OR "migratory pain" OR "migratory pains" OR "pains, migratory" OR "pain, radiating" OR "pains, radiating" OR "radiating pain" OR "radiating pains" OR "pain, splitting" OR "pains, splitting" OR "splitting pain" OR "splitting pains" OR "ache" OR "aches" OR "pain, crushing" OR "crushing pain" OR "crushing pains" OR "pains, crushing")
Fatigue Terms	#4	TITLE-ABS-KEY("fatigue" OR "lassitude" OR "tiredness")
Quality of Life Terms	#5	TITLE-ABS-KEY("quality of life" OR "life quality" OR "health-related quality of life" OR "health related quality of life" OR "hrqol")
Combined Search	#6	#1 AND #2 AND (#3 OR #4 OR #5)

Table S2. Characteristics of the studies included in this meta-analysis.

Study	Sample size (male/female)	Age (years)	Intervention	Intervention duration	Session duration	Frequency	Weekly time	Indicators
Arakaki et al., 2021	INT: 28 (0/28)<br>CON: 26 (0/26)	INT: 47.4 (9.0)<br>CON: 47.3 (8.7)	Swiss ball exercise	12 weeks	40 min	3	120 min	VAS (pain)<br>SF-36 (QOL)
Assumpção et al., 2017	INT1: 14 (0/14)<br>INT2: 16 (0/16)<br>CON: 14 (0/14)	INT1: 47.9 (5.3)<br>INT2: 45.7 (7.7)<br>CON: 46.9 (6.5)	INT1: Stretching exercise<br>INT2: Resistance exercise	12 weeks	40 min	2	80 min	VAS (pain)
Baptista et al., 2012	INT: 38 (0/38)<br>CON: 37 (0/37)	INT: 49.5<br>CON: 49.1	Belly dance	16 weeks	60 min	2	120 min	VAS (pain)<br>SF-36 (QOL)
Cedraschi et al., 2004	INT: 61 (NR)<br>CON: 68 (NR)	INT: 48.9 (9.7)<br>CON: 49.8 (9.8)	Exercise in pool and land	6 weeks	90 min	2	180 min	RPS (pain)

Collado-Mateo et al., 2017	INT: 42 (0/42) CON: 41 (0/41)	INT: 52.52 (9.73) CON: 52.47 (8.75)	Exercise game	8 weeks	60 min	2	120 min	EQ-5D-5L (QOL)
Demirbag et al., 2012	INT: 51 (4/47) CON: 51 (4/47)	NR	Education and exercise programs	4 weeks	40 min	1	40 min	VAS (pain)
Elgayar et al., 2025	INT: 37 (0/37) CON: 38 (0/38)	INT: 35.35 (3.12) CON: 36.57 (3.28)	Aerobic exercise	12 weeks	30 / 45 min	3	90 to 135 min	SF-12(QOL)
Ericsson et al., 2016	INT: 56 (0/56) CON: 49 (0/49)	INT: 22 to 64 CON: 22 to 64	Resistance exercise	15 weeks	60 min	2	120 min	MFI-20 (fatigue)
García-Martínez et al., 2012	INT: 12 (0/12) CON: 13 (0/13)	INT: 59.3 (4.8) CON: 58.6 (7.8)	Aerobic, strength, and stretching exercise	12 weeks	60 min	3	180 min	SF-36(QOL)
Garrido-Ardila et al., 2021	INT: 36 (0/36) CON: 33 (0/33)	INT: 56.06 (8.37) CON: 54.39 (8.2)	Core stability training	5 weeks	30 min	2	60 min	VAS (pain)

Haak et al., 2008	INT: 29 (0/29)<br>CON: 28 (0/28)	INT: 54 (9.4)<br>CON: 53.4 (8)	Qigong	7 weeks	90 min	NA	NA	VNS (pain)<br>WHOQOL-BR EF (QOL)
Jiao et al., 2019	INT: 29 (NR)<br>CON: 27 (NR)	INT: 48.9 (10.2)<br>CON: 53.5 (10.6)	Baduanjin	12 weeks	60 min	2	120 min	VAS (pain)<br>MAF (fatigue)<br>SF-36 (QOL)
Kolak et al., 2022	INT1:13 (0/13)<br>INT2: 13 (0/13)<br>CON:15 (0/15)	INT1: 48.3 (10)<br>INT2: 46 (11.2)<br>CON: 45.9 (7.6)	INT1: Aerobic and stretching exercises<br>INT2: Resistance and stretching exercises	12 weeks	NR	3	NR	VAS (pain)<br>SF-36 (QOL)
Larsson et all., 2015	INT: 56 (0/56)<br>CON: 49 (0/49)	INT: 50.81 (9.05)<br>CON: 52.1 (9.78)	Resistance exercise	15 weeks	25 min	2	50 min	VAS (pain)<br>SF-36 (QOL)
Latorre et al., 2013	INT: 42 (0/42)<br>CON: 30 (0/30)	INT: 52.4 (8.01)<br>CON: 50.39 (7.72)	Exercise in pool and land	24 weeks	60 min	3	180 min	VAS (pain)<br>SF-36 (QOL)

Latorre Román et al., 2015	INT: 20 (0/20)<br>CON: 19 (0/19)	INT: 51.7 (9.5)<br>CON: 50.25 (8.83)	Exercise in pool and land	18 weeks	60 min	3	180 min	VAS (pain)
Lynch et al., 2012	INT: 44 (NR)<br>CON: 45 (NR)	INT: 52.81(8.91)<br>CON: 52.13(8.56)	Qigong	8 weeks	45 to 60 min	7	315 to 420 min	NRS-PI (pain)<br>SF-36 (QOL)
Ribeiro et al., 2021	INT: 17 (0/17)<br>CON: 15 (0/15)	INT: 56<br>CON: 54	Whole-body vibration training	6 weeks	3 to 11 min	3	9 to 33 min	VAS (pain)
Rodríguez-Mansilla et al., 2021	INT1: 33 (0/33)<br>INT2: 31 (0/31)<br>CON: 29 (0/29)	NR	INT1: Active physical exercise<br>INT2: Qigong	4 weeks	45 min	2	90 min	VAS (pain)
Santen et al., 2002	INT1: 44 (0/44)<br>INT2: 38 (0/38)<br>CON: 27 (0/27)	INT1: 46.2<br>INT2: 44.4<br>CON: 42.8	INT1: Fitness training and education<br>INT2: Biofeedback training and education	24 weeks	INT1: 60 min<br>INT2: 30 min	2	INT1: 120 min<br>INT2: 60 min	VAS (pain)<br>VAS (fatigue)

Sañudo et al., 2011	INT: 18 (0/18)<br>CON: 20 (0/20)	INT: 55.48 (7.14)<br>CON: 56.15 (8.48)	Aerobic and muscle strength training	24 weeks	40 to 50 min	2	80 to 100 min	SF-36 s(QOL)
Sañudo et al., 2012	INT: 18 (0/18)<br>CON: 19 (0/19)	NR	Aerobic strength and stretching exercise	24 weeks	45 to 60 min	2	90 to 120 min	SF-36 (QOL)
Schachter et al., 2003	INT1:26 (0/26)<br>INT2: 29 (0/29)<br>CON: 31 (0/31)	INT1: 41.9 (8.57)<br>INT2: 41.3 (8.67)<br>CON: 42.5 (6.69)	INT1: Short bout of aerobic exercise<br>INT2: Long bout of aerobic exercise	16 weeks	INT1:<br>50 min<br>INT2:<br>40 min	4	INT1:<br>200 min<br>INT2:<br>160 min	VAS (pain)
Tomas-Carus et al., 2007	INT: 17 (0/17)<br>CON: 17 (0/17)	INT: 51(10)<br>CON: 51(9)	Exercised in warm water	12 weeks	60 min	3	180 min	SF-36 (QOL)
Villafaina et al., 2019	INT: 25 (0/25)<br>CON: 25 (0/25)	INT: 53.04 (9.56)<br>CON: 53.41(9.92)	Exercise game	24 weeks	60 min	2	120 min	VAS (pain)<br>EQ-5D-5L<br>(QOL)

Abbreviations: INT: intervention group; CON: control group; NR: no report; QOL: quality of life; VAS: visual analogue scale; RPS: regional

pain score; VNS: visual numerical scale; NRS-PI: numerical rating scale for pain intensity; MAF: multidimensional assessment of fatigue; MFI-20 scale: multidimensional fatigue inventory (4-20); SF-36 scale: short form health survey 36; SF-12: the 12-Item Short Form Health Survey; WHOQOL-BREF: the world health organization quality of life brief version; EQ-5D-5L: euroqol-5 dimensions-5 levels.

Table 3. GRADE summary of evidence.

Certainty assessment							No. of patients		Effect		Certainty	Importance
No. of studies	Study design	Risk of bias	Inconsistency	Indirectness	Imprecision	Other considerations	Exercise	Control	Relative (95% CI)	Absolute		
Pain (18)	RCT	serious[1]	serious[2]	no serious indirectness	no serious imprecision	reporting bias[3]	733	685	-	SMD 0.77 lower (1.06 to 0.48 lower)	⊕○○○ Very low	-
Fatigue (3)	RCT	serious[1]	serious[2]	no serious indirectness	no serious imprecision	reporting bias[3]	167	130	-	SMD 0.39 lower (0.73 to 0.05 lower)	⊕○○○ Very low	-
QOL (15)	RCT	serious[1]	no serious inconsistency	no serious indirectness	no serious imprecision	none	460	445	-	SMD 0.53 higher (0.39 to 0.66 higher)	⊕⊕⊕○ Moderate	-

Abbreviations: RCT: randomized controlled trial, SMD: standardized mean difference, CI: confidence interval, QOL: quality of life.

[1]Performance bias

[2]Substantial heterogeneity

[3]Funnel plot asymmetry

GRADE Working Group grades of evidence: ⊕⊕⊕⊕, High certainty: we are very confident that the true effect lies close to that of the estimate of the effect; ⊕⊕⊕○, Moderate certainty: we are moderately confident in the effect estimate: the true effect is likely to be close to the estimate of the effect, but there is a possibility that it is substantially different; ⊕⊕○○, Low certainty: our confidence in the effect estimate is limited: the true effect may be substantially different from the estimate of the effect; ⊕○○○, Very low certainty: we have very little confidence in the effect estimate: the true effect is likely to be substantially different from the estimate of effect.
